# Development of infectious clones of mungbean yellow mosaic India virus (MYMIV, *Begomovirus vignaradiataindiaense*) infecting mungbean [*Vigna radiata* (L.) R. Wilczek] and evaluation of a RIL population for MYMIV resistance

**DOI:** 10.1371/journal.pone.0310003

**Published:** 2024-10-22

**Authors:** Nikki Kumari, Muraleedhar S. Aski, Gyan Prakash Mishra, Anirban Roy, Harsh Kumar Dikshit, Shipra Saxena, Manju Kohli, Bikash Mandal, Subodh Kumar Sinha, Dwijesh Chandra Mishra, Md Firoz Mondal, Ranjeet Ranjan Kumar, Atul Kumar, Ramakrishnan M. Nair

**Affiliations:** 1 Division of Genetics, Indian Agricultural Research Institute, New Delhi, India; 2 Division of Plant Pathology, Indian Agricultural Research Institute, New Delhi, India; 3 National Institute for Plant Biotechnology, New Delhi, India; 4 Agricultural Bioinformatics, Indian Agricultural Statistics Research Institute, New Delhi, India; 5 Division of Biochemistry, Indian Agricultural Research Institute, New Delhi, India; 6 Division of Seed Science and Technology, Indian Agricultural Research Institute, New Delhi, India; 7 World Vegetable Center, South Asia, ICRISAT Campus Patancheru, Hyderabad, India; Jeju National University, REPUBLIC OF KOREA

## Abstract

Yellow mosaic disease (YMD) is a major constraint for the low productivity of mungbean, mainly in South Asia. Addressing this issue requires a comprehensive approach, integrating field and challenge inoculation evaluations to identify effective solutions. In this study, an infectious clone of *Begomovirus vignaradiataindiaense* (MYMIV) was developed to obtain a pure culture of the virus and to confirm resistance in mungbean plants exhibiting resistance under natural field conditions. The infectivity and efficiency of three *Agrobacterium tumefaciens* strains (EHA105, LBA4404, and GV3101) were evaluated using the susceptible mungbean genotype PS16. Additionally, a recombinant inbred line (RIL) population comprising 175 lines derived from Pusa Baisakhi (MYMIV susceptible) and PMR-1 (MYMIV resistant) cross was developed and assessed for YMD response. Among the tested *Agrobacterium tumefaciens* strains, EHA105 exhibited the highest infectivity (84.7%), followed by LBA4404 (54.7%) and GV3101 (9.80%). Field resistance was evaluated using the coefficient of infection (CI) and area under disease progress curve (AUDPC), identifying seven RILs with consistent resistant reactions (CI≤9) and low AUDPC (≤190). Upon challenge inoculation, six RILs exhibited resistance, while RIL92 displayed a resistance response, with infection occurring in less than 10% of plants after 24 to 29 days post inoculation (dpi). Despite some plants remaining asymptomatic, MYMIV presence was confirmed through specific PCR amplification of the MYMIV coat protein (AV1) gene. Quantitative PCR revealed a very low relative viral load (0.1–5.1% relative fold change) in asymptomatic RILs and the MYMIV resistant parent (PMR1) compared to the susceptible parent (Pusa Baisakhi). These findings highlight the potential utility of the developed infectious clone and the identified MYMIV-resistant RILs in future mungbean breeding programs aimed at cultivating MYMIV-resistant varieties.

## Introduction

Mungbean [*Vigana radiata* (L.) R. Wilczek] is one of the important grain legume crops, grown mainly in South Asian countries, which offers many nutritional and economic benefits [1]. It is grown on about 5.13 m ha in Indian subcontinent with an annual production of 3.09 mt [2]. Although the yield potential of mungbean is nearly 2.5–3.0 t/ha, its average productivity staggers around 0.6 t/ha [2, 3]. Yellow mosaic disease (YMD) is a major constraint for the low productivity of mungbean in Indian subcontinent. The estimated annual yield loss due to YMD in mungbean, soybean, and black gram crops is around 300 million USD [4]. However, significant economic impact of YMD, with losses accounting for up to an 85% reduction in yield specifically in India [5].

Based on sequence identity, four different species of begomoviruses belonging to the family *Geminiviridae* have been reported to cause YMD in mungbean and other crops of Leguminosae. As per the recent taxonomic release, these species are: *Begomovirus vignaradiatae* (mungbean yellow mosaic virus or MYMV), *Begomovirus vignaradiataindiaense* (mungbean yellow mosaic India virus or MYMIV), *Begomovirus loniceramusivi* (horsegram yellow mosaic virus or HgYMV) and *Begomovirus dolichoris* (dolichos yellow mosaic virus or DYMV). Additionally, other viruses such as kudzu mosaic virus and velvet bean mosaic virus also infect legumes. Among these, MYMIV is predominant in the northern, central, and eastern parts [6], while MYMV in southern and western regions of India [7, 8]. Like other begomoviruses, these species are also transmitted by whiteflies (*Bemisia tabaci* Genn.) [9].

Begomoviruses have twinned (geminate) incomplete icosahedral particle (18×30 nm), harboring one or two circular ssDNA genomic components, designated as DNA-A and DNA-B (each of size ca. 2.7kb). Functionally, DNA-A is regulating encapsidation, replication, and transcription; while DNA-B is involved in the regulation of intra- and inter-cellular movement of proteins- needed for infection, host range determination, symptoms expression, and nuclear localization [10, 11]. Both DNA-A and DNA-B contain a non-coding region (ca. 200bp) having motifs regulating host range determination, replication, and gene expression [12]. Due to high recombination rate, and error prone replication strategy adopted by begomoviruses, several sequence variants of begomovirus species have been reported from different legume crops in the Indian subcontinent [13, 14]. To ascertain the evaluation of resistance in a particular crop, thus characterization of a particular sequence variant present in a particular location is essentially a prerequisite.

The YMD management is mainly through vector control by insecticide spray, which is neither economically viable nor environmentally safe. Additionally, prolonged insecticides use has eventually augmented the development of multi-fold resistance in whiteflies against different insecticides. Thus, the use of resistant varieties is most efficient, economical, and environment-friendly way of YMD management. The major challenge faced by the researchers in the development of MYMIV-resistant genotypes is the identification of true resistant lines from any population and also the lack of a reliable screening protocol combining both field and artificial screening for MYMIV expression.

Most of the studied screening procedures relied upon either natural disease pressures or are based on laboratory screening [15, 16]. In the absence of a uniform and robust screening technique, no reliable results could be obtained [17]. The field screening mainly depends upon the whitefly transmission efficiency, which varies with host genotypes and vector biotypes. Therefore, field screening for YMD response is not considered a very reliable method [18]. Agro-inoculation of plants using infectious clone is considered as the other alternative method for screening of resistance, however, preference of different Agrobacterium strains with different genotypes may also varies [19, 20]. A robust screening method thus include field screening, whitefly-based challenge inoculation and final confirmation through inoculation of agroinfectious clones [15].

Recombinant inbred lines (RILs) are highly homozygous plants with known pedigree, and are considered best for the studying the quantitative trait expression across season and locations. Therefore, these are considered best for the screening of genotypes showing resistance to YMD. With this background, the present study aimed to characterize the begomovirus isolate exists in the experimental field and develop an agro-infectious clone of the current isolate. Furthermore, we utilized a robust screening strategy involving field evaluation, whitefly transmission, and agroinoculation to confirm resistance in the parental line and identify resistant RIL genotype(s).

## Material and methods

### Construction of RIL population and field evaluation methodologies

An intra-specific RIL population was derived by crossing a MYMIV susceptible genotype Pusa Baisakhi (as female parent) with a MYMIV resistant genotype PMR1 (as male parent). The F_1_ was grown which resulted in F_2_ (310 No) population. The subsequent generations were maintained through single seed descent method for the creation of RILs. The RIL population (F_6:7_ and F_7:8_) consisting of 175 lines and its parents were evaluated (under open field conditions) for YMD reaction during *kharif-*2020 (F_7_) and *kharif-*2021 (F_8_) at Indian Agricultural Research Institute (IARI), New Delhi (hot spot for YMD) in randomized complete block design (RCBD) with two replications. The experimental field was located at the latitude (28.64°N), longitude (77.15°E) and altitude of 228 m AMSL (above mean sea level).

The PMR1 is resistant to MYMIV, while Pusa Baisakhi is an agronomically superior high-yielding variety, but susceptible to MYMIV. The plants were grown in a 4-m row (40 plants/row) and 30×10 cm spacing was kept between row-to-row and plant-to-plant, respectively. Spreader row technique was used to ascertain consistent and sufficient viral load; and PS16 (MYMIV susceptible) was sown after every 05-rows and also around the plots. The selection of the five plants per row was done on random basis from each row of the RIL population, to ensure unbiased sampling for the overall MYMIV response per row. Furthermore, MYMIV incidence was evaluated when at least 80% of the spreader row plants showed MYMIV infection. Prescribed cultivation practices were followed, except insecticides spray to maintain the natural whiteflies population.

The disease incidence was recorded four times at 15 d intervals starting from 15 d after sowing (DAS) till 60 DAS, while disease severity was recorded at 60 DAS. Based on the percent severity, severity grades (0–4), and response values (0–1) were assigned (Table 1) [21] and coefficient of infection (CI) was calculated [15]. Using CI values, disease reaction is classified in 6 groups, namely highly resistant (HR), resistant (R), moderately resistant (MR), moderately susceptible (MS), susceptible (S), and highly susceptible (HS). The area under disease progress curve (AUDPC) for MYMIV infestation was measured using the following formula [22]:

$$Y=\sum_{i=1}^{n}[(x_{i}+x_{i})/2](t_{i+1}-t_{i})$$

Y is the AUDPC, *x*_*i*_ is the disease incidence of the i^th^ evaluation and *x*_*i*+1_is the disease incidence of the i+1^st^ evaluation. (*t*_*i*+1_-*t*_*i*_) is the number of d between two evaluations. The disease progress curve was created using R package ver.4.2.2: (https://www.rdocumentation.org/packages/agricolae/versions/1.3-5/topics/audpc).

**Table 1:** Disease scoring scale.

Symptoms	Severity grade	Response value (RV)^b^	Coefficient of Infection (CI)^a^	Disease reaction (DR)
Symptoms absent	0	0	0–4	Highly resistant
Very mild symptoms up to 25% leaves	1	0.25	5–9	Resistant
Appearance of symptoms in 26–50% leaves	2	0.50	10–19	Moderately resistant
Appearance of symptoms in 51–75% leaves	3	0.75	20–39	Moderately susceptible
Severe disease infection in more than 75% leaves	4	1	40–69	Susceptible
			70–100	Highly susceptible

^a^ CI = Percent disease incidence [(No. of infected plant(s)/ total no. of plants) **×**100] **×** Response value.

^b^ Response value is quantitative transformed value of percent severity range in 0 to 1 scale. Percent severity range is calculated based on cumulative value of all the disease evaluation parameters (Source: Bag et al., 2014)

### Source of virus-infected plant material

**Fig 1:**
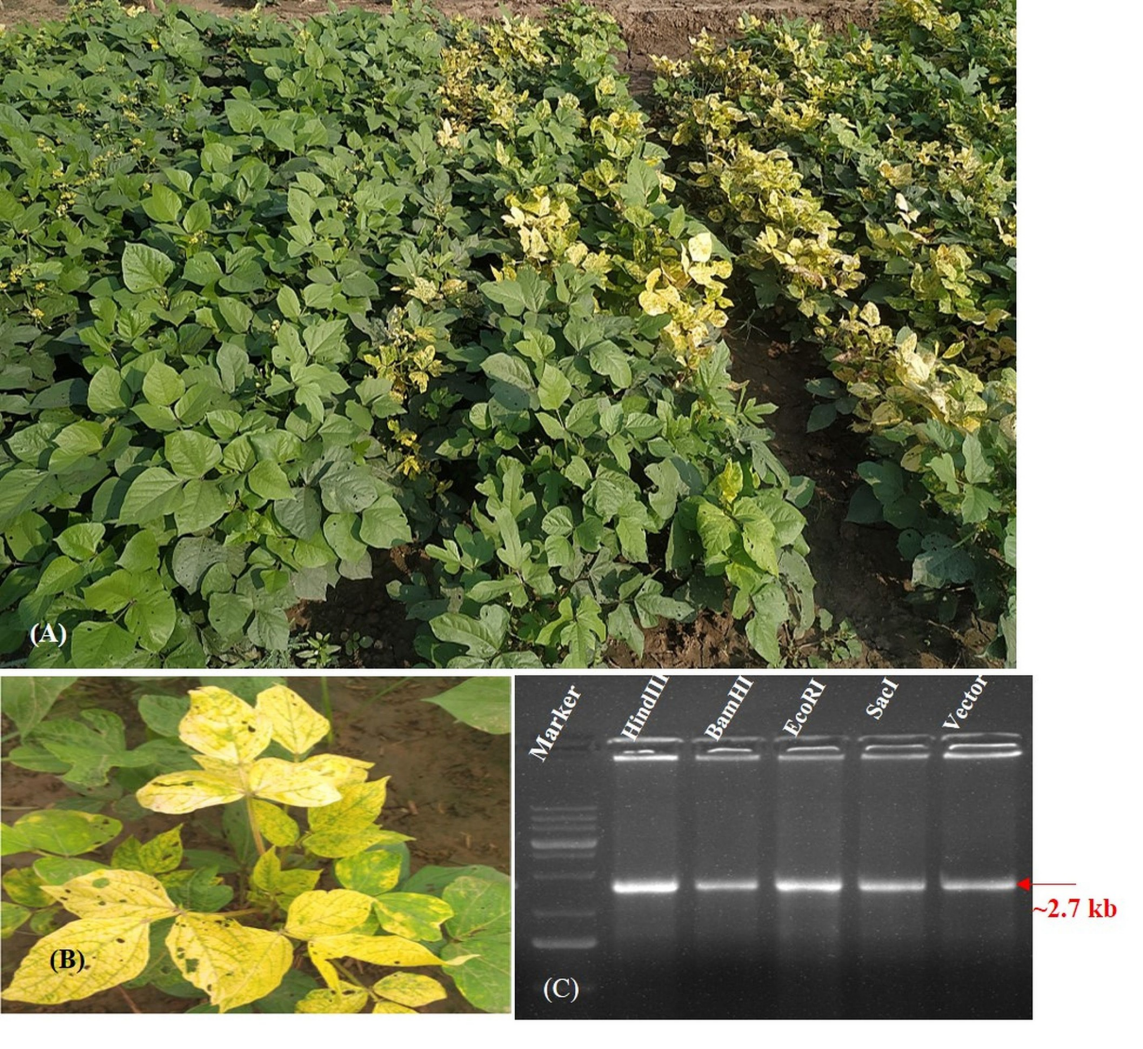
**(A)** Yellow mosaic disease reaction of a mungbean RIL (resistant and susceptible) along with a susceptible check under open field conditions of North India (New Delhi). **(B)** Closeup of the *Mungbean yellow mosaic India virus* infection. **(C)** Restriction digestion of RCA products extracted from naturally infected plant leaves. Where, DNA marker is on extreme left, while digestive enzymes are denoted at the top.

The YMD incidence in RILs was studied twice during *kharif* (2020 and 2021) at IARI, New Delhi under open field conditions. For the identification of predominant begomovirus strain prevalent in the experimental field conditions, genomic DNA was isolated from the leaves of 12 symptomatic RILs from an open field (Fig 1), using CTAB method [23]. As previous studies indicated presence of MYMIV in mungbean crop from northern India [24], PCR amplification was carried out using a pair MYMIV species specific primers (BM-925F: 5’-AGG TGT CCC TAC CAA CAT-3’; BM-926R: 5’-CCA TGG ATT GTT CCT TAC AA-3’) designed from AV1 gene of MYMIV, with an expected amplicon size of 510 bp. PCR reaction include preheating (94°C; 2–3 min), followed by denaturation (94°C; 30-sec), annealing (53°C; 45-sec), extension (72°C; 40-sec) and was repeated for 35 cycles followed by final extension (72°C; 10 min). The amplified product was resolved on agarose gel (1%) and positive sample was used as a source plant for characterization of the complete genomic components of MYMIV isolate present in the field condition. To ascertain the presence of MYMV, in any of these samples, a MYMV species specific primer pairs BM-931F (5’-AAG TGT CCC TGC CAG CGC-3’) and BM-932R (5’-CGG GTT GAA GAA AGC ACT GCT-3’), designed from the AV1 and AC2 gene, respectively, were utilized, with an expected amplification size of ca. 925 bp. The PCR conditions were same as described earlier except for annealing temperature which was 60°C.

### Rolling circle amplification and cloning of full-length viral genomes

Full-length MYMIV genome was amplified through ɸ-29 DNA polymerase based rolling circle amplification (RCA) using the exo-nuclease resistant random hexamers following the standard method [25]. Briefly, total DNA (50ng), 1X ɸ-29 enzyme buffer, 10 μM exo-resistant random primers and 2 mM dNTP were added to a tube and incubated at 95°C for 5 min for denaturation and annealing of the primers. After cooling the reaction mixture for 2 min on ice, 0.02 unit of pyrophosphatase and 5 units of ɸ-29 DNA polymerase were added and incubated at 30°C for 18 followed by inactivation of the enzyme at 65°C for 10 min. The concatemeric RCA product was linearized by incubating RCA product (4.0 μl) with RE like *Hind*III, *Eco*RI, *Sac*I, and *Bam*HI (Thermo Scientific FastDigest), and resolved on 0.8% agarose gel. *Bam*HI digested ca.2.7kb fragment was purified using QIAquick Gel Extraction Kit (QIAGEN, Valencia, CA, USA), ligated into *Bam*HI-digested linear pUC18 vector and transformed into *E*.*coli* DH5α cells. The recombinant clones were identified through colony PCR and restriction digestion of the plasmids. Further DNA-A and DNA-B recombinant colonies were identified by single digestion with *Hind*III and *Noc*I and also by double digestion with *Bam*HI and *Pst*I. Confirmed recombinant clones of DNA-A and DNA-B were sequenced by outsourcing.

### Sequence and recombination analysis

The sequences of monomeric clones of DNA-A and DNA-B were analysed through BLAST (http://blast.ncbi.nlm.nih.gov). The sequence alignment, percentage identity matrix, and predicted amino acid sequences were generated (BIOEDIT ver.7.0), multiple sequence alignment and phylogenetic analysis were done (MEGA X) and phylogenetic trees were constructed (using maximum parsimony algorithm). The DNA-A and DNA-B sequences of MYMIV available at GenBank were used for comparative studies (S1 and S2 Tables) using standard nomenclature [26]. Recombination analysis was performed using Recombination Detection Program 4 (RDP4) through RDP, BootScan (BS), Max Chi (MC), GENECOV (GC), Chimera (Chi), 3Seq and Si Scan (SS) using other begomoviruses causing YMD [27].

### Construction of partial tandem repeat

The partial tandem repeat (PTR) construct for the MYMIV isolate under study was developed in sequential steps. Initially, recombinant plasmids of full-length DNA-A and DNA-B were subjected to double-digestion using *Bam*HI and *Xba*I (for DNA-A) and *Bam*HI and *Eco*RI (for DNA-B). *Bam*HI/*Xba*I digested 1.1 kb fragment of DNA-A (0.4 mer) and *Bam*HI/*Eco*RI digested 1.5 kb fragment of DNA-B (0.5 mer) contained the origin of replication and sub-cloned into pCAMBIA2300 to generate recombinant pCAMBIA-DNA-A-0.4 and pCAMBIA-DNA-B-0.5 clones. Further, the 2.7 kb fragment (*Bam*HI fragment,1 mer) of DNA-A and DNA-B were rescued by digestion with *Bam*HI/*Bgl*I and *Bam*HI/*Sac*I, respectively, from their pUC18 clones. The 1mer fragment of both DNA-A and DNA-B were subsequently cloned into the *Bam*HI site of pCAMBIA-DNA-A-0.4 and pCAMBIA-DNA-B-0.5 plasmid, respectively. The orientation of the tandem repeats constructs was confirmed by restriction digestion with *Xba*I and *Eco*RI for DNA-A and DNA-B, respectively.

### Agro-inoculation

PTR constructs of MYMIV isolate under study was mobilized separately to three different strains of *Agrobacterium tumefaciens* (EHA105, GV3101 and LBA4404) by freeze-thaw method and the transformants were selected under rifampicin (25 mg/mL) and kanamycin (50 mg/mL). To confirm the infectivity, agroinoculation was performed on 2-d old sprouted seeds of PS16 (susceptible) by slightly removing the seed coat and pricking the epicotyl using fine needle [9]. Pricked seeds were then kept with the overnight grown cultures of *A*. *tumefaciens* (EHA105, GV3101, and LBA4404) separately containing the PTR constructs of DNA-A and DNA-B. For screening the RILs, *Agrobacterium* culture was prepared using (i) semi-solid culture method, and (ii) liquid agro-culture method [28]. These cultures were used for the infection of pin-pricked seeds of the RIL population and their parents in the epicotyl region for 1h, 2h, and overnight. The seeds inoculated with blank pCAMBIA2300 were used as mock control. The inoculated seeds were incubated (28°C), washed, and sown in pots. Agroinoculated plants were maintained in an insect-proof growth chamber under controlled conditions (at 25–26°C; 80–90% RH; 16:8h light:dark duration) and symptoms were observed periodically.

### Detection of MYMIV in agro-inoculated plants

Total DNA was isolated from the young leaves of both asymptomatic, and symptomatic plants and viral DNA was checked through PCR using primers for DNA-A (BM-925F: 5’-AGG TGT CCC TAC CAA CAT-3’; BM-926R: 5’-CCA TGG ATT GTT CCT TAC AA-3’) and DNA-B (BV1-61F: 5’-TTT GTG CAA TAC CCT GTT CG-3’; BV1-62R: 5’-TGT CCC AAG GAC TTT GAA GC-3’). Reaction mixture consisted of DNA (400–500 ng), dNTP (0.5 mM), primers (0.5 μM), and *Taq* polymerase (1.0-unit). Amplification conditions for DNA-A were the same as mentioned above, while for DNA-B initial denaturation (94°C; 2 min) was followed by first cycle at 94°C for 30 sec, 55°C for 45 sec, and 72°C for 1 min and was repeated for 35 cycles and final extension was done at 72°C for 10 min.

### Relative quantification of MYMIV using real-time PCR

Genomic DNA was extracted from the leaves of agro-inoculated symptomatic and asymptomatic RILs, parents, and healthy control plants. qPCR was performed in three technical and biological replicates using primers AV1-F (5’TAC TGG GAA GGT TTG GAT GG3’) / AV1-R (5’TCA CGC AGA TCG TTC TTC AC3’) and AC1-F (5’ATT ATC TTT GCG GCC ATT TG3’)/AC1-R (5’TGG TGG GGA TAC CAC CTT TA3’), which generate 174bp and 186bp amplifications, respectively. The reaction mixture (10μL) consisted of 1.0μL template DNA, 5.0μL Power SYBR® Green Master Mix (Applied Biosystems, Foster City, Calif., USA), 1.0μL each forward and reverse primers (0.5 μM), and 2.0μL nuclease-free water. The amplification conditions include: denaturation (95°C; 2 min), followed by 40 cycles of 95°C for 10s, 55°C for 30s, and 72°C for 30s. Finally, a melting curve was made from temperature 60 to 95°C, with an increment of 0.5°C every 5.0s. GraphPad Prism ver.9.5.0 (https://www.graphpad.com/scientific-software/prism/) was used to analyze the one-way ANOVA and bar graph. *Vigna mungo* actin (ACT) was used as an endogenous normalization measure [29] and the relative viral load was calculated using ΔCt method [30].


$$\Delta {\text{Ct}}_{\text{av}}{=[\text{Ct}}_{\text{av}}\;\left(\text{virus}\right){‐\text{Ct}}_{\text{av}}\;\left(\text{end}\right)]$$

Where, end: endogenous reference gene; Ct_av_: Average of three technical replicates for both virus and endogenous reference gene.

Fold change (FC) of the viral DNA load between different samples was calculated:

$$\text{FC}=2^{-\Delta \Delta {\text{Ct}}_{\text{av}}}$$

Where: ΔΔCt_av_ compares the viral load in different samples.

Relative quantification [31] which does not require construction of a standard curve was used to determine the begomovirus titre to differentiate resistant and susceptible cultivar [32, 34].

### Whitefly inoculation of plant

Resistant (PMR-1) and susceptible (Pusa Baisakhi) parents were inoculated (as control) artificially using viruliferous whiteflies and tested using PCR for the presence of virus. For this, symptomatic Pusa Baisakhi was used as inoculum source for adult whiteflies (10 whiteflies/plant) [35] and given 24h acquisition access period (AAP), then released on healthy plants at 2-leaf stage (24h inoculation access period). Thereafter, whiteflies were killed by spraying Imidacloprid (0.3 mL/L water) and plants were kept in an insect-proof glasshouse for 5 weeks for symptom development.

## Results

### Detection of begomovirus associated with mungbean

For YMD infection, the mungbean RILs screening was done under natural field conditions (Fig 1) at IARI, New Delhi (a hotspot region for MYMIV). The differentiation between MYMIV and MYMV, solely based on field symptoms is very challenging, as both viruses induce similar symptoms. Thus, PCR amplification of 12 randomly selected susceptible RILs (infected) and healthy plants (as negative controls) using MYMV and MYMIV specific primers showed MYMIV-specific amplification (500 bp) in the infected samples, and no amplification of MYMV-specific fragments (925 bp) (S1 Fig). PCR assay also recorded absence of alpha-satellite and beta-satellite in the infected mungbean samples. Following amplification of the virus genome by rolling circle amplification (RCA) [25], the resulting product was digested with *Hind*III, *Eco*RI, *Sac*I, and *Bam*HI restriction enzymes, which yielded a ca. 2.7 kb fragment, similar to that of the unit genome length of begomoviruses. *Bam*HI-digested RCA product was cloned into pUC18 vector. With restriction digestion, two colonies were identified which represented the DNA-A and DNA-B clones of MYMIV isolates from New Delhi. Complete nucleotide sequence data of DNA-A and DNA-B are submitted to the NCBI GenBank databases (https://www.ncbi.nlm.nih.gov/WebSub/) with accession numbers OP850814 and OP850815, respectively.

### Molecular characterization of the virus through cloning and sequencing

**Fig 2:**
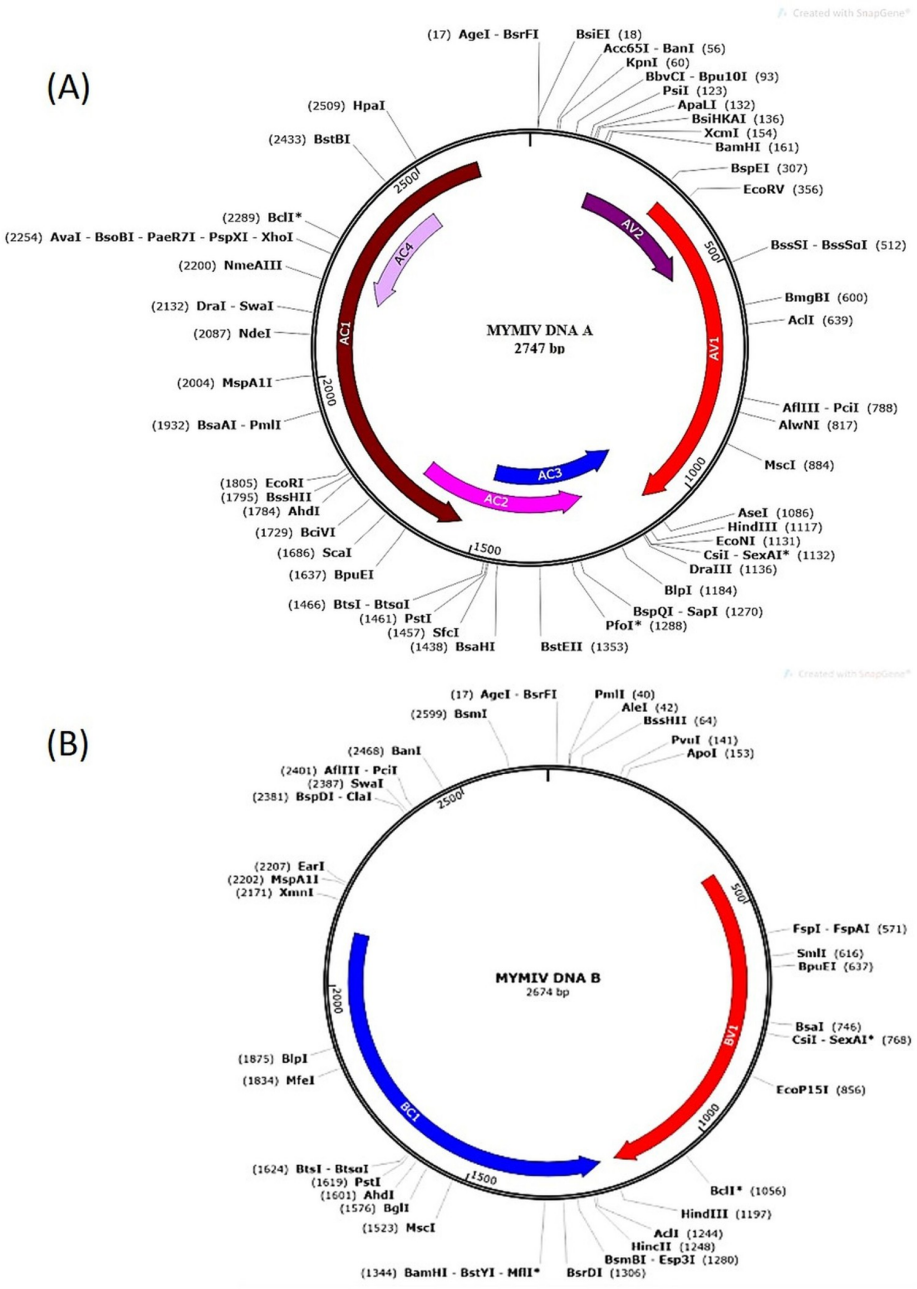
Annotated complete genome map of DNA-A and DNA-B of begomovirus displaying viral ORFs with respective nucleotide coordinates and unique restriction enzymes spanning the genome.

The complete DNA-A (2747nt) and DNA-B (2674nt) sequence (GenBank accession number OP850814 and OP850815, respectively) of MYMIV showed typical old world begomoviruses genome organization. ORF analysis (https://www.ncbi.nlm.nih.gov/orffinder/) of DNA-A showed two ORFs in the sense strand (AV1 and AV2) and five in the antisense strand (AC1, AC2, AC3, AC4, and AC5), which are separated by an intergenic region (Fig 2). Maximum sequence identity was recorded with an Indian MYMIV isolate (98.7%; MW917145) and was followed by Pakistan (>95%; AM950268.1 and AY271895.1) and Nepal MYMIV isolates (94.9%; JN543395). The AV1 and AV2 were very close with French bean MYMIV isolate (FN794200), while AC1, AC2, and AC3 showed closeness with urdbean MYMIV isolate (MW917145). AC4 revealed >99% identity with MYMIV isolates of French bean and soybean (KP779635 and MH32445). Highest amino acid identity was recorded with the Indian MYMIV isolates in all the 6 ORFs of DNA-A (S3 Table).

Pairwise sequence identity of DNA-B revealed maximum sequence identity with an Indian urdbean MYMIV isolate (95.8%), followed by MYMIV isolates (95%) of pigeonpea and French bean (KX363948, KP779634, respectively). BV1 and BC1 showed maximum amino acid sequences identity with MYMIV isolates. Also, high sequence identity was recorded with MYMIV isolates of Pakistan (FM202442), Nepal (JN543396), and Oman (MK757224). Low similarity was recorded between DNA-B of mungbean and HgYMV (68.1%), DoYMV (55.10%), and ToLCNDV (45.10%). Overall, DNA-B of mungbean revealed high similarity to MYMIV isolates (S1 Table). Motif scan predicted various kinases, while C1 protein had an ATP binding motif. The ATPase motif is required for nicking and ligation of origin of replication. cNLS mapper predicted the presence of nuclear localization signal in V1 and C1 proteins of MYMIV (S2 and S4 Tables). Based on a pairwise nucleotide sequence identity matrix of DNA-A and DNA-B, a heat map was generated (S2 Fig).

### Phylogenetic analysis

**Fig 3:**
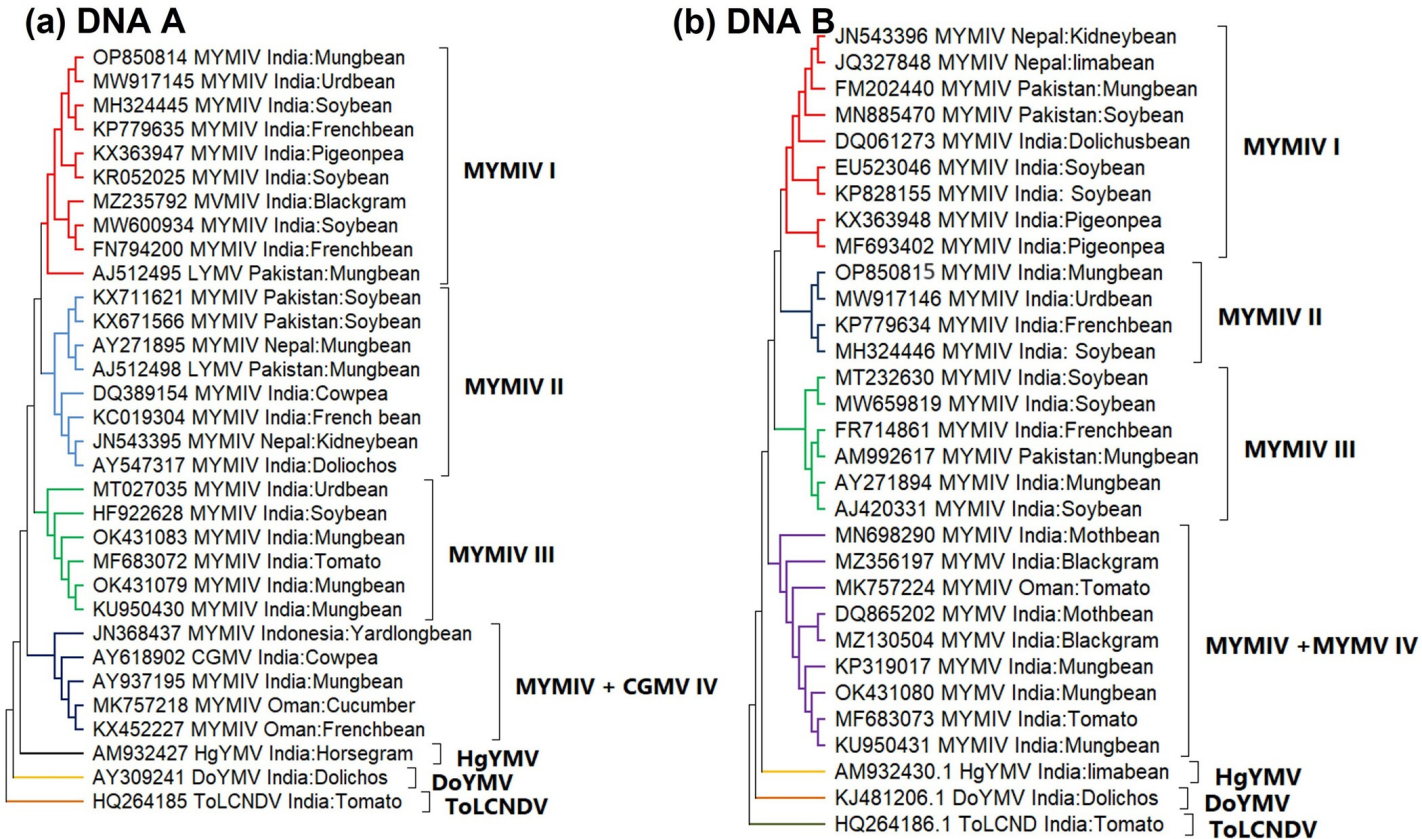
Phylogenetic relationship of MYMIV of **(a)** DNA-A and **(b)** DNA-B with other related begomoviruses infecting different pulses. The dendrogram was constructed using 1000 bootstrap replicates through Neighbour Joining method.

Phylogenetic analysis of DNA-A of mungbean with other legume isolates showed distinct clustering of MYMIV, HgYMV, and DoYMV, with that of non-leguminous hosts (ToLCNDV) (Fig 3A). The phylogenetic tree based on DNA-A revealed proximity to urdbean isolates from Northern India, as well as to MYMIV isolates from India, Pakistan, and Nepal, rather than isolates from Oman and Indonesia (Fig 3A).

The phylogenetic tree of DNA-B of mungbean showed maximum similarity with the urdbean MYMIV isolate; which could be divided into four subgroups viz., MYMIV ӏ, MYMIV ӏӏ, MYMIV ӏӏӏ and MYMIV + MYMV IV (Fig 3B). Group I (MYMIV I) comprises isolates from India, Pakistan, and Nepal. Group II (MYMIV II) includes isolates solely from India (DNA-B of mungbean). Group IV (MYMIV + MYMV IV) contains isolates of both MYMIV and MYMV. Interestingly, HgYMV, DoYMV, and ToLCNDV are independently positioned within the same sub-cluster.

RDP4 based recombination analysis of DNA-A and DNA-B revealed that DNA-A has a recombination breakpoint between 1450 and 1859 nt position. Software predicted the major parent as an isolate of MYMIV (KX363947) infecting pigeonpea, while the minor parent was an isolate of MYMIV (FN794200) infecting French bean, both reported from India (S5 Table). Recombination analysis of DNA-B of the present isolate showed recombination break point between 185 and 346 nt position (in the intergenic region), and in this case the major parent was an isolate of MYMIV reported from Pakistan infecting mungbean (AM992617) and minor parent was an isolate of MYMIV reported from India infecting soybean (MW659819) (S6 Table).

### Infectivity of cloned MYMIV constructs

**Fig 4:**
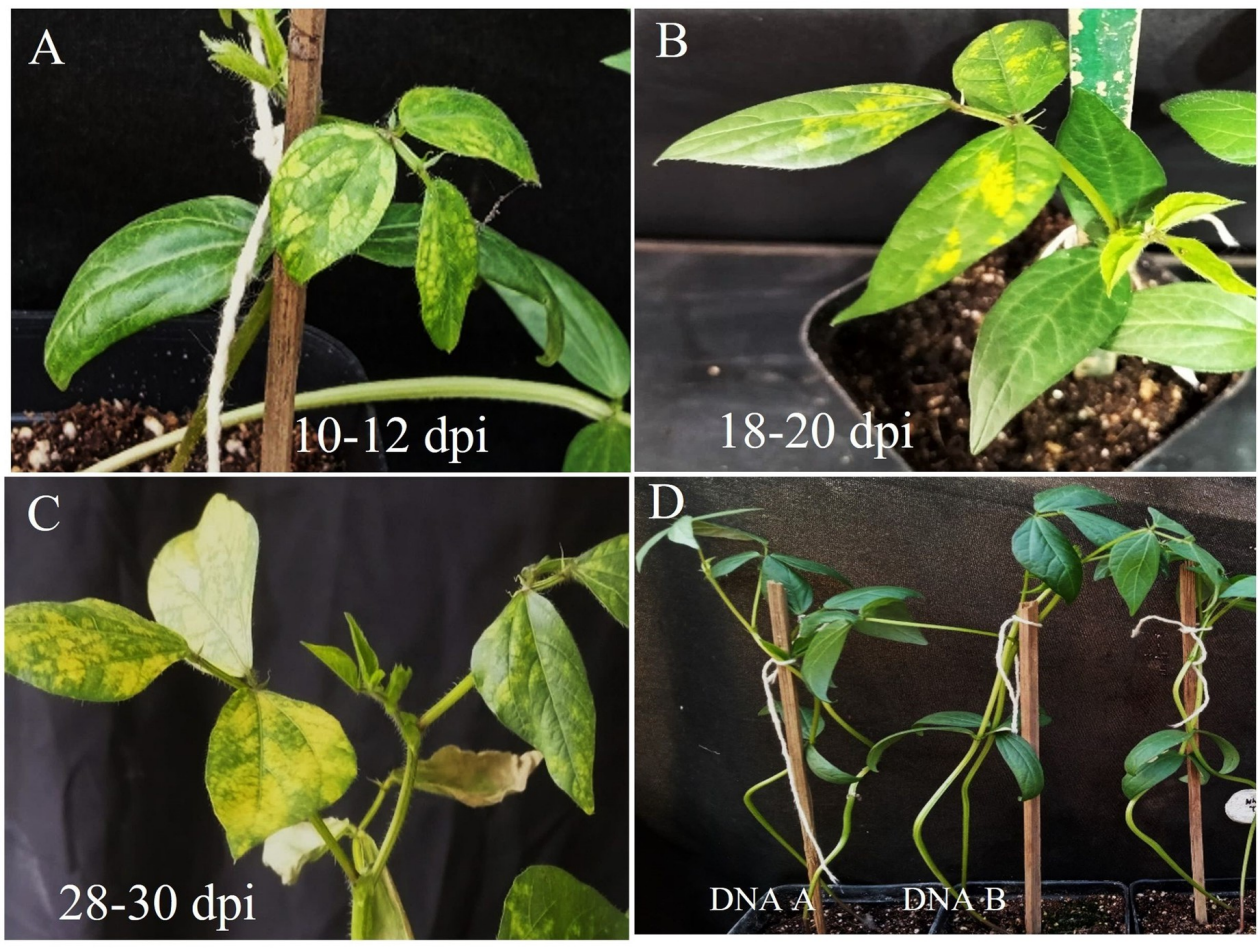
Symptom progression in *Vigna radiata* cv. PS-16 represented at three different stages: (A) 10–12 dpi, (B) 18–20 dpi, and (C) 28–30 dpi. (D) Individual inoculations with either DNA A or DNA B did not show any symptoms, similar to the mock-inoculated plant.

Upon agroinoculation of the PTR constructs of DNA-A and DNA-B clones of the present isolate initial symptoms were observed on 10^th^ days post inoculation (dpi) using a highly susceptible mungbean genotype (PS16) to MYMIV. Within 20 dpi entire lamina became bright yellow, while complete chlorosis was recorded at 28–30 dpi (Fig 4A–4C). In the case of mock inoculation (pCAMBIA-2300 without insert) or when DNA-A and DNA-B were used independently, no disease symptom was recorded (Fig 4D).

The infectivity of three *Agrobacterium* strains using pin-pricked sprouted seeds showed EHA-105 (77.1–84.7%) giving more pronounced symptoms over and LBA-4404 (47–54.7%) and GV-3101 (9.52–9.8%) during 8–12 dpi (S3 Fig and Table 2). Thus, EHA-105 was used for further experiments to screen the RILs. Interestingly, there was no significant impact of time duration when the germinated sprouts were incubated with agriculture for 1 or 2 h, however, germination rate and infectivity (%) of overnight incubation were quite low (Table 2). Liquid culture was found more effective for screening of large mungbean population, as semi-solid culture was more time-consuming and labor-intensive.

**Table 2:** Infectivity of *Vigna radiata* cv. PS-16 using PTR constructs of MYMIV.

Strain	Biological replicates	Incubation period (h)			Average infectivity (%)	Method		Symptom appearance (d)
		1h	2h	Overnight		Semi-solid (SS)	Liquid culture (LC)	
EHA-105	Exp-I	15/16	17/19	7/11	84.7	SS	-	10–12
	Exp-II	11/13	10/12	6/10	77.1	-	LC	10–12
LBA 4404	Exp-I	8/14	7/16	¼	47.0	SS	-	10–12
	Exp-II	11/16	9/19	3/7	54.7	-	LC	10–12
GV 3101	Exp-I	2/18	3/20	0/13	9.80	SS	-	8–10
	Exp-II	1/14	2/17	1/11	9.52	-	LC	8–10

Exp: Experiment, h: Hours, d: Days

### Field screening for virus resistance

**Fig 5:**
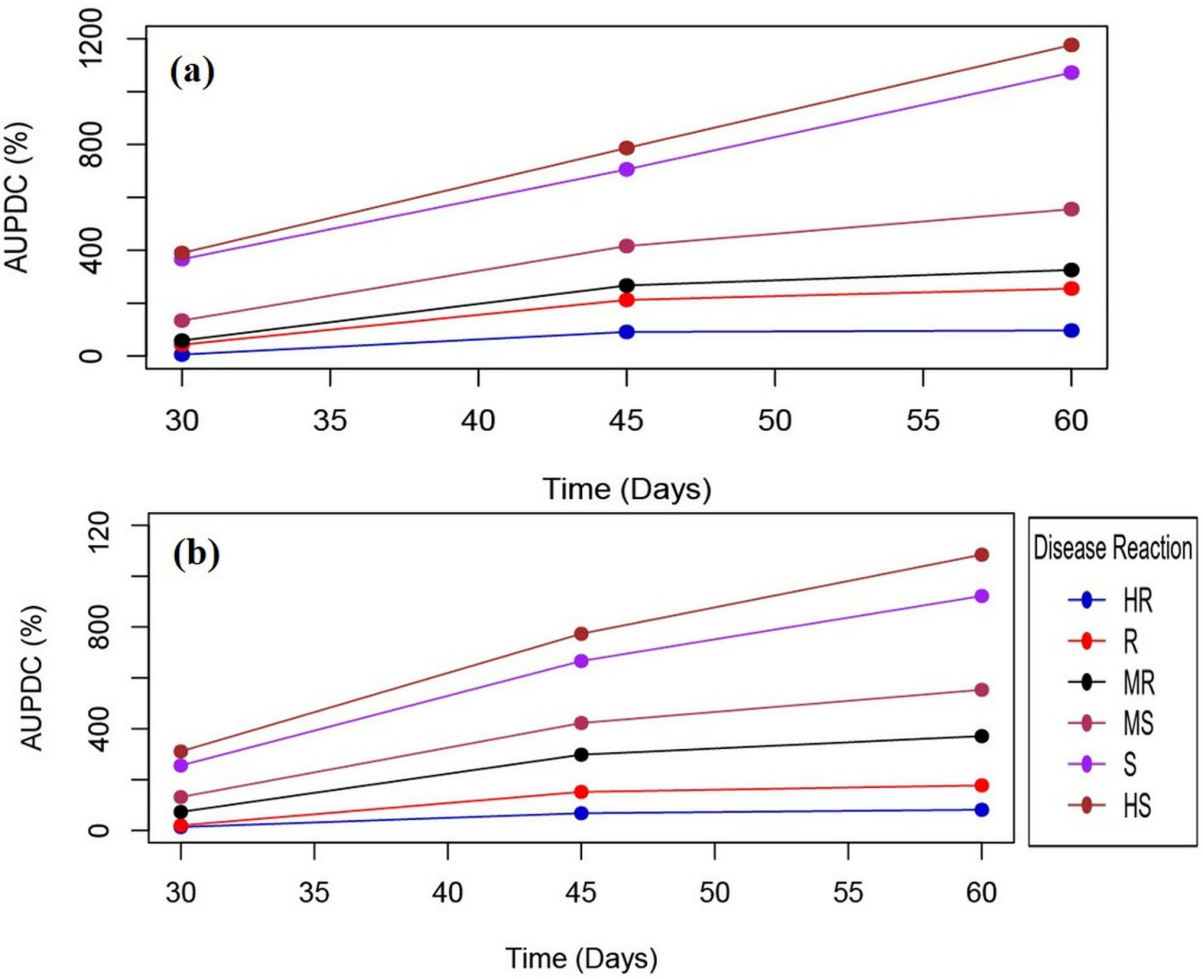
MYMIV disease progress curve of different disease reactions during (a) *kharif* 2020, (b) *kharif-*2021. Disease reactions are indicated with different colors. The X-axis represents the time duration as 30, 45, and 60d; while Y-axis represent AUDPC values (%).

The frequency histogram in S4 Fig illustrates the distribution of MYMIV disease over a span of two years. Analysis reveals that the distribution pattern of this trait across the two years conforms to a normal distribution. Consequently, it can be inferred that the trait is subject to quantitative inheritance. The disease severity (DS) and percentage disease incidence (PDI) of 175 RILs (*kharif* 2020 and *kharif* 2021) showed maximum lines as moderately susceptible (MS, 43%), followed by susceptible (S, 20%) and highly susceptible (HS, 4%) disease reaction (S7 Table). Under resistant category, 03 plants were highly resistant (HR; CI≤3.9), 18 as resistant (R; CI≤8.7), and 37 as moderately resistant (MR; CI≤18.8). Field screening (*kharif* 2020 and *kharif* 2021 from July to November) confirmed the response of RILs to YMD (S8 Table). The susceptible genotypes (including spreader rows) showed very high CI (86.66) and AUDPC values (222.36) during 2-year screening, indicating the presence of significant virus inoculum. Of all, 07 RILs showed HR or R reactions (CI = 3.4–8.9) during both the years, indicating nearly uniform and stable resistance response (Table 3). In addition, R-lines expressed delayed disease onset, lower severities, and also slower pace of disease progression. Interestingly, the disease progression curves for all the six disease reaction categories showed variations in the progress of disease over time during both the years (Fig 5).

**Table 3:** MYMIV disease response of parents, susceptible check, and a sub-set of RILs under natural epiphytotic conditions during *kharif*-2020 and 2021.

S. No.	RILs/parents/check	*Kharif* (2020–21)			*Kharif* (2021–22)		
		CI	AUDPC	DR	CI	AUDPC	DR
1.	**RIL10**	**7.27**	**147.77**	**R**	**6.09**	**115.38**	**R**
2.	**RIL13**	**5.30**	**148.13**	**R**	**4.04**	**105.12**	**HR**
3.	RIL16	17.50	262.13	MR	13.92	264.20	MR
4.	**RIL21**	**6.39**	**151.77**	**R**	**8.10**	**121.10**	**R**
5.	RIL28	14.55	278.87	MR	15.62	362.69	MR
6.	RIL33	15.39	236.20	MR	12.09	240.26	MR
7.	RIL59	19.93	404.83	MR	12.74	325.72	MR
8.	RIL63	14.23	212.77	MR	17.09	301.36	MR
9.	**RIL92**	**7.69**	**104.78**	**R**	**8.69**	**176.10**	**R**
10.	RIL107	13.73	289.76	MR	17.10	482.14	MR
11.	**RIL123**	**3.93**	**56.96**	**HR**	**3.43**	**78.91**	**HR**
12.	RIL126	17.50	212.64	MR	18.86	188.66	R
13.	**RIL131**	**8.09**	**201.45**	**R**	**7.57**	**123.67**	**R**
14.	**RIL163**	**8.23**	**289.95**	**R**	**4.38**	**187.68**	**R**
15.	RIL172	16.13	241.94	MR	12.37	185.62	MR
16.	RIL173	11.21	336.21	MR	12.63	189.53	MR
17.	PMR-1	1.00	7.00	HR	0.00	0.00	HR
18.	Pusa Baisakhi	100.00	2289.65	HS	96.87	2300.00	HS
19.	Susceptible check (PS16)	100.00	2167.43	HS	100	2356.74	HS

Where, shaded RILs showed consistent HR/R response over two years; CI: Co-efficient of infection; AUDPC: Area under disease progress curve; DR: Disease reaction

### Confirmation MYMIV disease reaction in RILs through agro-inoculation

Agro-inoculation of PMR-1, Pusa Baisakhi, control, and mock-inoculated plants using infectious clone showed typical yellow mosaic symptoms only in Pusa Baisakhi. Agroinculation on seven RILs (No 10, 13, 21, 92, 123, 131, and 163) that exhibited field resistance (R/HR) during both kharif-2020 and kharif-2021 confirmed their resistance to MYMIV, except RIL92. The RIL92 showed bright yellow spots after 24–29 dpi in <10% of plants (S5 Fig). Within 10–12 days post-inoculation (dpi), severe YMD symptoms were recorded in 84.44% of the susceptible check (PS-16). Additionally, within 18–20 dpi, severe YMD symptoms appeared in 71.11% of the susceptible parent Pusa Baisakhi (Table 4).

**Table 4:** YMD reaction of parents, RILs, and a susceptible check when agro-inoculated with the infectious MYMIV (NDI).

RILs/ parents/ check	No. of plants infected/ No. of plants inoculated			Average infectivity (%)	Incubation period (d)	Symptoms	DR
	Exp.1	Exp.2	Exp.3				
RIL10	0/15	0/15	0/15	0	Nil	Nil	HR
RIL13	0/15	0/15	0/15	0	Nil	Nil	HR
RIL21	0/15	0/15	0/15	0	Nil	Nil	HR
RIL92	3/15	0/15	1/15	6.6	24–29	Coalesced bright yellow specks or spots	MR
RIL123	0/15	0/15	0/15	0	Nil	Nil	HR
RIL131	0/15	0/15	0/15	0	Nil	Nil	HR
RIL163	0/15	0/15	0/15	0	Nil	Nil	HR
PMR-1	0/15	0/15	0/15	0	Nil	Nil	HR
PS16 (SC)	12/15	13/15	13/15	84.44	10–12	Yellow mosaic or chlorosis of leaves	HS
Pusa Baisakhi	11/15	12/15	9/15	71.11	18–21	Yellow mosaic or chlorosis of leaves	HS

DR: Disease reaction, HR: Highly resistant, MR: Moderately resistant, HS: Highly susceptible; SC: Susceptible check

### Relative viral DNA accumulation in susceptible and resistant plant

**Fig 6:**
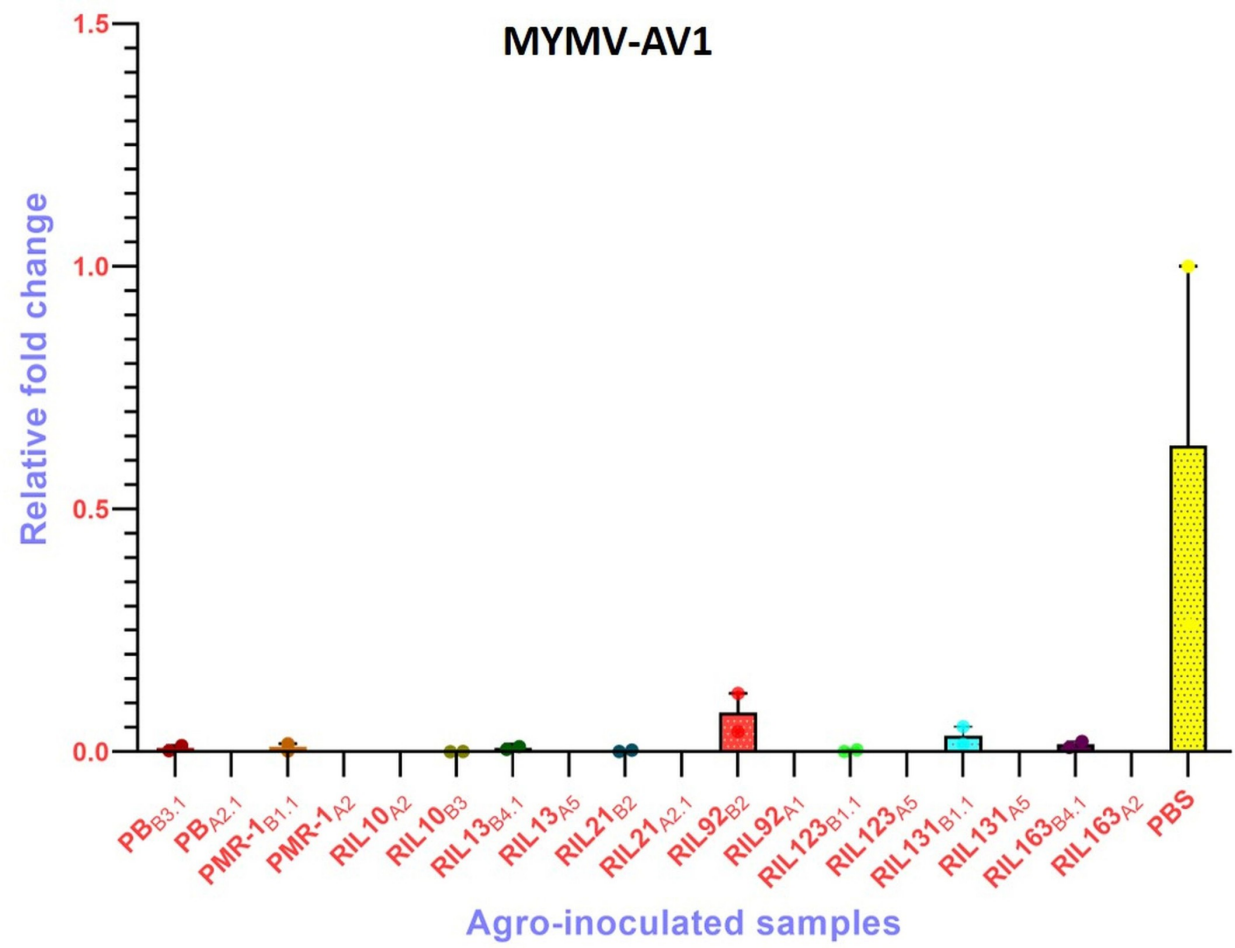
Relative quantification of MYMIV in resistant RILs and their parents using qPCR. Where, bars represent the relative fold change of the virus in the samples normalized to the amount of plant DNA represented by the endogenous *Vigna mungo* actin (ACT) gene. Vertical lines on each bar represent SEM. Subscripts A and B represent the absence and presence of a band, respectively; followed by their respective sample numbers. PB: Pusa Baisakhi; PBS: Pusa Baisakhi (Infected).

In Pusa Baisakhi, sporadic yellow mosaic symptoms first appeared at 10 dpi, and by 30 dpi, the plants become fully infected, while PMR-1 and 6-resistant RILs showed no symptom (S5 Fig). Interestingly, some of the test plants did not express any symptoms (Asymptomatic) but MYMIV presence was confirmed through PCR amplification of the coat protein gene (AV1) (S6 Fig). Further, qPCR also confirmed the relative viral load in the asymptomatic plants. Mean C_t_ values of MYMIV-infected symptomatic samples were in the range of 9.6±0.11 to 30.4±1.81 (CV<9%) (S9 Table). However, viral DNA in Pusa Baisakhi (susceptible) was recorded significantly high (P<0.001, R^2^ = 0.88). Infected asymptomatic plants (PMR-1_B1.1_, PB_B3.1_, RIL10_B3_, RIL13_B4.1_, RIL21_B2_, RIL123_B1.1_, RIL131_B1.1_, and RIL163_B4.1_) showed 0.001 to 0.051-fold increase in viral DNA load, which indicated that the accumulation of viral DNA is significantly reduced in those lines. In contrast, RIL92_B2_ showed very high (0.12-fold change) viral DNA concentration (Fig 6). The presence of very low levels of viral DNA in the 06 resistant RILs and PMR-1 was correlated with the lack of clear YMD symptoms. This could be due to constraints in virus multiplication, which result in low levels of CP formation. The amount of viral DNA and subsequent CP production plays a critical role in determining virus infectivity and symptom expression. However, other factors, such as plant defense mechanisms and gene expression, may also contribute to the observed resistance and lack of symptoms.

### Whitefly transmission to confirm the resistance in parent and asymptomatic lines

The back inoculation of MYMIV from asymptomatic PMR-1 (resistant parent) to the uninfected Pusa Baisakhi and PMR-1 plants did not show any yellow mosaic symptoms (S7 Fig). Afterward, when MYMIV infected Pusa Baisakhi plant was used for the back inoculation of a healthy Pusa Baisakhi plant, yellow specks are observed on the leaves 25 dpi (S7 Fig). This means that the asymptomatic plants do not carry sufficient viral load to cause any infection in healthy plants including the susceptible genotype.

## Discussion

Host resistance can be affected by factors like climatic conditions, availability of viruliferous whiteflies, and inoculum pressure. Thus, it is challenging to gauge the YMD severity response based solely on either PDI score or percent disease severity (PDS) [36–38]. To overcome this, CI was computed using both PDI and PDS [15] and YMD response of RILs were assessed under field conditions. AUDPC was used to assess the cumulative progression of YMD, which varied significantly across susceptible and resistant genotypes [39, 40]. The AUDPC for both years showed very slow disease progression in HR, R, and MR genotypes until 30d, and reached a plateau after 45d. Whereas in HS, S, and MS genotypes, the rate of disease progression continued even after 45d. Such patterns were also recorded in blackgram when infected with YMV [15]. The slow disease progression was commonly reported for plant-virus interactions, wherein resistant plants may exhibit some initial symptoms but gets recovered by forming young asymptomatic leaves [41].

Infection with mixed or multiple viruses, have previously been reported to cause severe damage in urdbean and mungbean crops [42, 43]. However, Asia II 1 and Asia I were reported as the prevalent whitefly biotype in northern and central parts of India. While Asia II 7 is the most prominent whitefly biotype in the Delhi region [44]. Also, the host range of soybean isolate of MYMIV, etiology of YMD of black gram, soybean, and cowpea are extensively studied [45]. A few studies also report the association of MYMIV with YMD [9, 46, 47]. However, detailed molecular studies of viruses infecting mungbean under North Indian field conditions are lacking [24].

The study employed PCR-based detection followed by RCA and sequencing to elucidate the prevalent virus in IARI fields, New Delhi, revealing MYMIV as the predominant virus. RCA facilitated the characterization of the full MYMIV genome into two nearly 2.7 kb DNA molecules [31, 46, 48]. BLAST analysis demonstrated high sequence identity (>98% for DNA-A and 96% for DNA-B) with MYMIV isolates from urdbean in India. Phylogenetic analysis grouped both DNA-A and DNA-B with an urdbean isolate from Northern India, and also revealed a close relationship with MYMIV isolates from neighbouring countries like Pakistan and Nepal. Recombination analysis unveiled the evolutionary dynamics of the present isolate, suggesting intraspecific recombination events.

In addition to PCR-based detection and sequencing, the establishment of infectious clones of plant viruses is crucial for comprehensively characterizing viral pathogens. Infectious clones allow researchers to study various aspects of viral biology, including replication, host range, movement, and pathogenicity [32–34, 49].

Till date, most of the studies used MYMIV isolated from soybean, cowpea, and blackgram for testing its infectivity [6, 46, 47]. Only a few reports are there from India where mungbean isolates are used for the development of infectious clone [28, 50]. Thus, agro-infectious clone developed from mungbean isolates (North India) is an invaluable asset to support breeding for resistance in mungbean.

Till date, mostly the transformation efficiency of different *Agrobacterium* strains [51, 52] and standardisation of different agroinoculation parameters [9, 19, 28, 45] were done to achieve maximum infection efficiency. However, the efficacy of different *Agrobacterium* strains for its infectivity has not been tested thoroughly for mungbean. A few studies also reported the use of EHA105 in mungbean [28, 46] and blackgram [45]. However, present study represents the first comparison of the infectivity of three *A*. *tumefaciens* strains, ultimately confirming EHA105 as the most effective strain for screening a large number of mungbean genotypes for MYMIV reaction. Agroinoculation offers the advantage of developing uniform disease symptoms, facilitating easier scoring compared to natural infection [53]. The highest percent infectivity (93–100%) was observed in sprouted seeds and seedlings of mungbean cv Maha Gujarat using the agroinoculation method [28]. However, variations in infectivity percentages were noted among different studies and genotypes. For example, a MYMIV susceptible mungbean genotype PS16 was agroinoculated with MYMV showed an infectivity rate of only 36% [54], while studies on agroinoculation of MYMIV in mungbean reported infectivity frequencies of 48% and 23%, respectively [45, 55]. In the present study, an infectivity rate of 77.1–84.7% was achieved in a MYMIV susceptible mungbean genotype PS16 using the agroinoculation method. Thus, the variation in results may be attributed to the use of different genotypes. Interestingly, in our previous study, a positive correlation between MYMIV resistance (in RILs) and two agronomic traits viz., higher trichome density and SPAD value were recoded [56]. Thus, these traits need to be studied in some more genotypes and populations to find their relation with the imposition of MYMIV resistance in mungbean.

This study not only characterize an isolate of MYMIV, but also the infectious clones generated in the study have been used to confirm the resistance of few RILs that showed field resistance. Often begomovirus infection does not produce symptomatic expression and that leads to ambiguity for identification of resistance source. In this study the parents and RILs are initially screened under natural condition and those consistently showed resistance response, were further evaluated through agroinoculation. Those genotypes showed resistance even after agroinoculation, the viral load was also detected to be low. Further, the whitefly back inoculation study revealed that in the resistant parent and RILs the viral load was not sufficient to be transmitted by whitefly. Thus, using a robust and comprehensive strategy accommodating both field and artificial screening, the confirmed resistance source have been achieved.

## Conclusions

The biggest impediment for the YMD management is proper screening of genotypes for the identification of true resistance source to MYMIV under North Indian conditions [57]. This study involved, thorough screening, combining repeated field screening and agro-inoculation of a RIL population against MYMIV. The infectivity and efficiency of *A*. *tumefaciens* strains could identify EHA105 as the best strain. The six RILs showing consistent resistant reaction can be used as a source to develop MYMIV-resistant varieties by targeted crossing program.

## Supporting information

S1 Fig Viral gene specific amplification of susceptible RILs. (A) MYMIV specific amplification (∼500bp) using AV1 gene specific primers (BM925F & BM926R). (B) No amplification observed by MYMV gene specific primers (AV1-Fwd. & AC1-Rev.). Where, M: Marker; -ve: Negative control; 1–12: No. of susceptible RILs.
(JPG)

S2 Fig Heat map depicting nucleotide identity based on complete DNA-A genome and DNA B. (JPG)

S3 Fig Effect of different strains on virus infection, (A) EHA 105, (B) LBA 4404, (C) GV 3101.
(JPG)

S4 Fig Histograms illustrating the frequency distribution for MYMIV resistance in RIL population during (A) 2020 and (B) 2021.
(JPG)

S5 Fig Response of PMR-1, Pusa Baisakhi, and 07 RILs (RIL10, 13, 21, 92, 123, 131, 163) to MYMIV under agro-inoculation at 65 dpi. (JPG)

S6 Fig Viral gene-specific amplification (AV1; 174bp) of agro-inoculated plants (PMR-1, Pusa Baisakhi, 07-RILs). Where, M: Marker (100 bp); C: -ve control using genomic DNA from a healthy plant; Lanes 1–6: DNA from PMR-1, Pusa Baisakhi, RIL10, RIL13, RIL21, RIL123, RIL92, RIL131 and RIL163; Lanes 1–7: DNA from genotypes Pusa Baisakhi (Asymptomatic line: PBA).
(JPG)

S7 Fig Back-inoculation source as asymptomatic leaves of PMR-1. (a) PMR1- Resistant response, (b) Pusa Baisakhi–asymptomatic response. 2. Back-inoculation source was symptomatic leaves of Pusa Baisakhi. (c) Pusa Baisakhi–Susceptible response.
(JPG)

S1 Table Sequence identity of the complete DNA A genome, different ORFs at nucleotide (Nt) and amino acid (aa) level of begmovirus clone with other related begmoviruses (analysed by Mega X version 10.2.6 and BioEdit version 7.2). (DOCX)

S2 Table Sequence identity of the complete DNA B genome, different ORFs at nucleotide (Nt) and amino acid (aa) level of begomovirus clone with other related begomoviruses (analyzed by Mega X version 10.2.6 and BioEdit version 7.2). (DOCX)

S3 Table List of functional motifs identified in the amino acid sequence of different ORFs in MYMIV (identified by Motif scan tool). (DOCX)

S4 Table Identification of nuclear localization signal (NLS) in different ORFs of MYMIV clone. (DOCX)

S5 Table Recombination analysis of DNA A of Mungbean yellow mosaic India virus using RDP 4.101 tool showing intraspecific recombination. (DOCX)

S6 Table Recombination analysis of DNA B of Mungbean yellow mosaic India virus using RDP 4.101 tool showing intraspecific recombination. (DOCX)

S7 Table Preliminary screening and grouping of 175 RILs based on their reaction to MYMIV in *Kharif* 2020. (DOCX)

S8 Table Advance field screening and grouping of 175 RILs based on their reaction to MYMIV in *Kharif* 2021. (DOCX)

S9 Table The cycle threshold (C_t_) values of replicate assays for MYMIV. (DOCX)
