# Supplementary figures and images for: Development of infectious clones of mungbean yellow mosaic India virus (MYMIV, Begomovirus vignaradiataindiaense) infecting mungbean [Vigna radiata (L.) R. Wilczek] and evaluation of a RIL population for MYMIV resistance
Source: PLoS One. 2024 Oct 22;19(10):e0310003. doi: 10.1371/journal.pone.0310003 (PMC11495560; doi:10.1371/journal.pone.0310003)

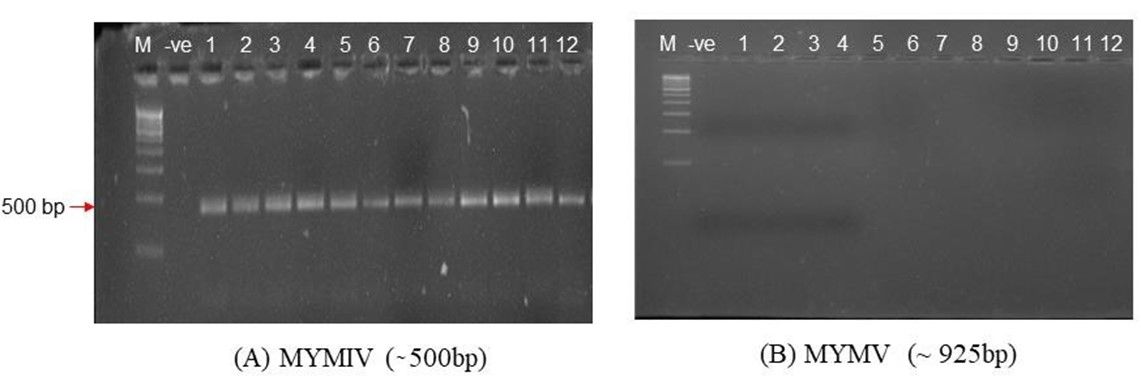

Supplement: S1 Fig — (A) MYMIV specific amplification (∼500bp) using AV1 gene specific primers (BM925F & BM926R). (B) No amplification observed by MYMV gene specific primers (AV1-Fwd. & AC1-Rev.). Where, M: Marker; -ve: Negative control; 1–12: No. of susceptible RILs. (JPG) [file pone.0310003.s001.jpg]

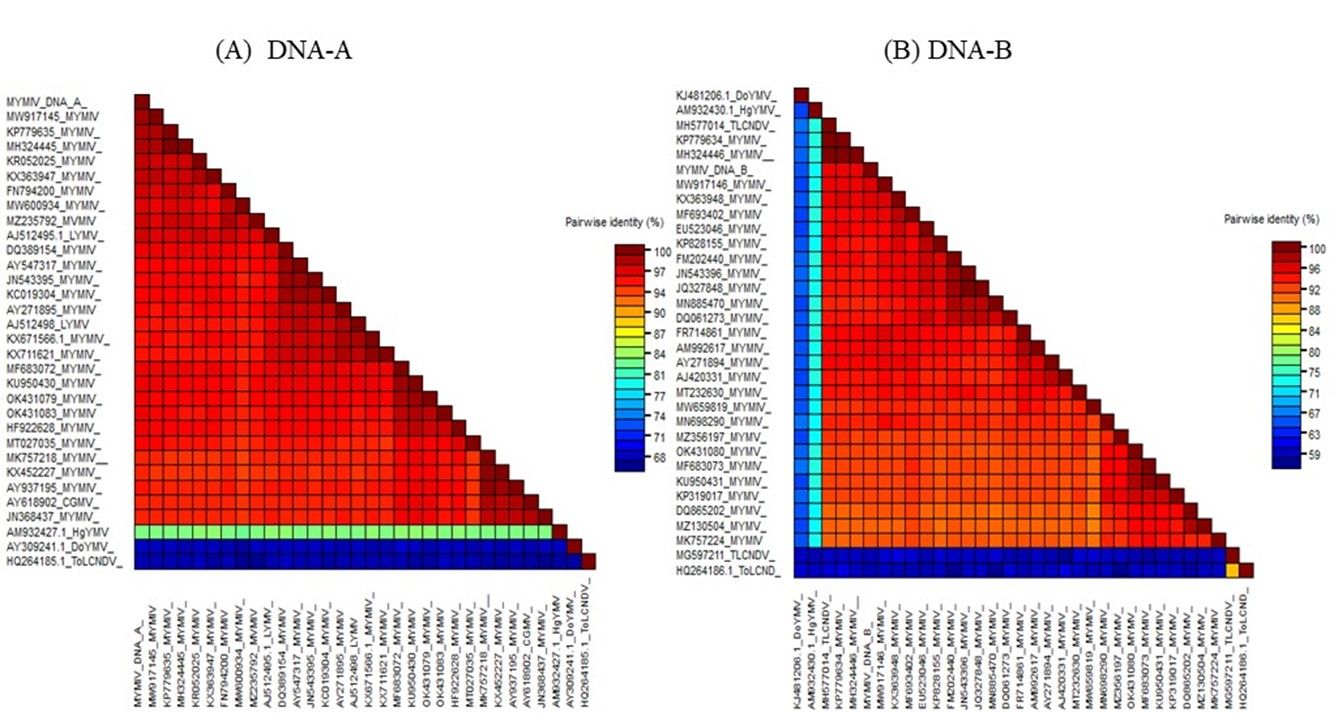

Supplement: S2 Fig — (JPG) [file pone.0310003.s002.jpg]

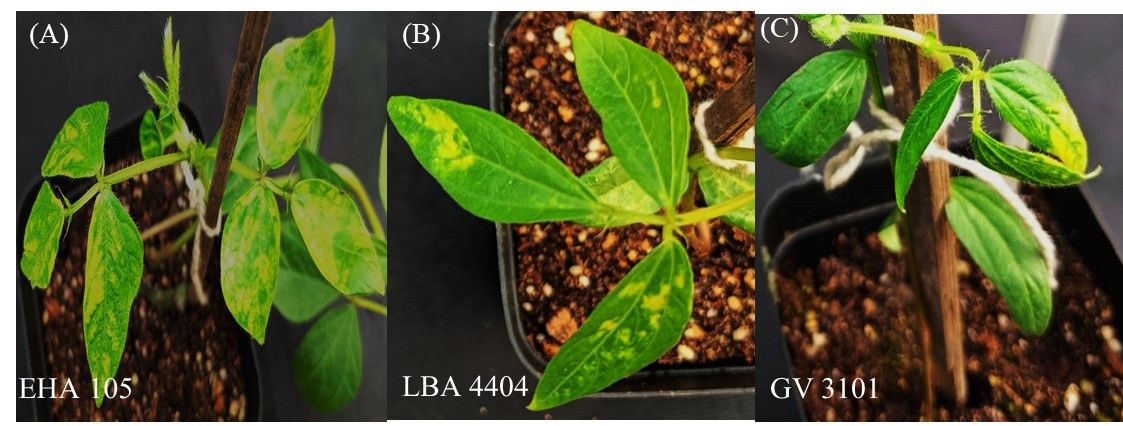

Supplement: S3 Fig — Effect of different strains on virus infection, (A) EHA 105, (B) LBA 4404, (C) GV 3101. (JPG) [file pone.0310003.s003.jpg]

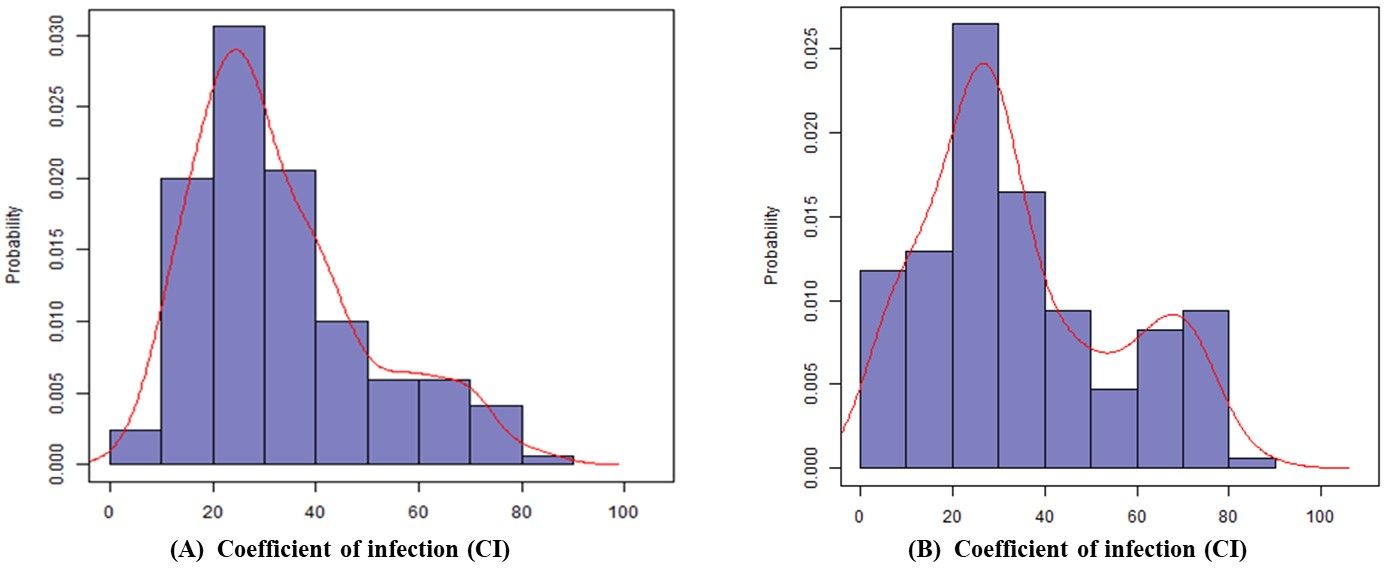

Supplement: S4 Fig — Histograms illustrating the frequency distribution for MYMIV resistance in RIL population during (A) 2020 and (B) 2021. (JPG) [file pone.0310003.s004.jpg]

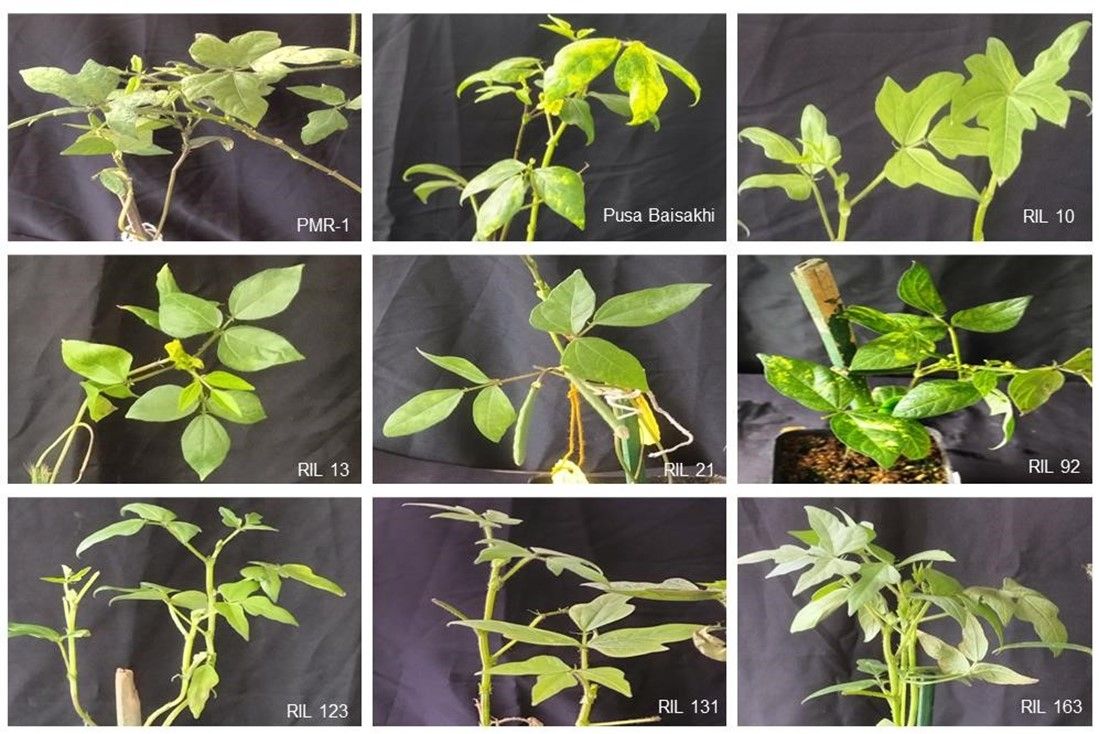

Supplement: S5 Fig — (JPG) [file pone.0310003.s005.jpg]

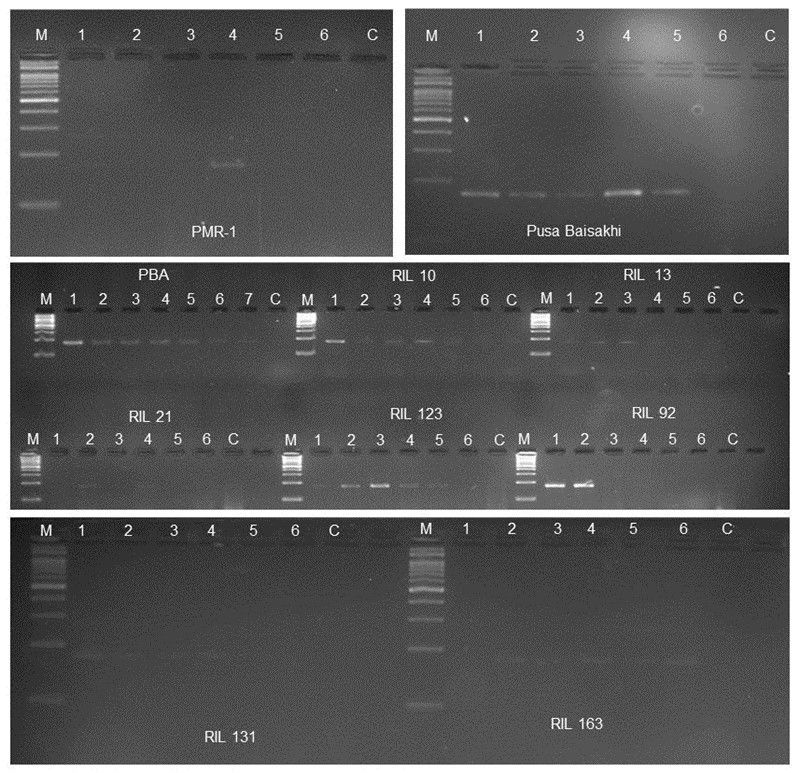

Supplement: S6 Fig — Where, M: Marker (100 bp); C: -ve control using genomic DNA from a healthy plant; Lanes 1–6: DNA from PMR-1, Pusa Baisakhi, RIL10, RIL13, RIL21, RIL123, RIL92, RIL131 and RIL163; Lanes 1–7: DNA from genotypes Pusa Baisakhi (Asymptomatic line: PBA). (JPG) [file pone.0310003.s006.jpg]

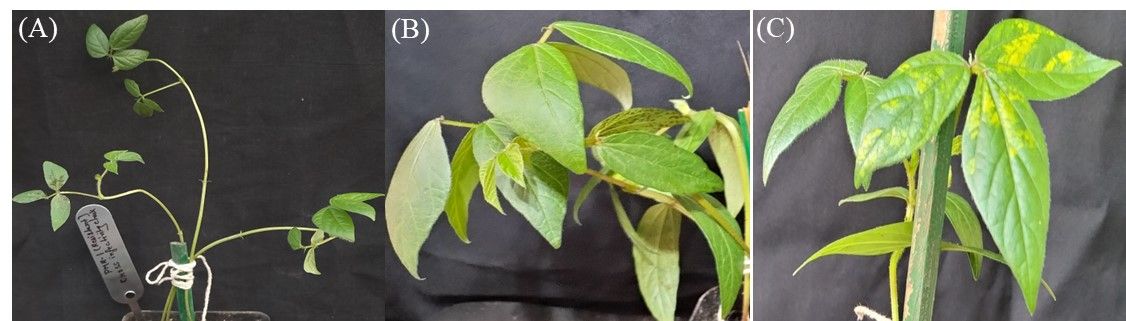

Supplement: S7 Fig — (a) PMR1- Resistant response, (b) Pusa Baisakhi–asymptomatic response. 2. Back-inoculation source was symptomatic leaves of Pusa Baisakhi. (c) Pusa Baisakhi–Susceptible response. (JPG) [file pone.0310003.s007.jpg]
