# Supplementary material for: Development of infectious clones of mungbean yellow mosaic India virus (MYMIV, Begomovirus vignaradiataindiaense) infecting mungbean [Vigna radiata (L.) R. Wilczek] and evaluation of a RIL population for MYMIV resistance
Source: PLoS One. 2024 Oct 22;19(10):e0310003. doi: 10.1371/journal.pone.0310003 (PMC11495560; doi:10.1371/journal.pone.0310003)
Supplement: S1 Table — (DOCX) [file pone.0310003.s008.docx]

**Table S1. Sequence identity of the complete DNA A genome, different ORFs at nucleotide (Nt) and amino acid (aa) level of begmovirus clone with other related begmoviruses (analysed by Mega X version 10.2.6 and BioEdit version 7.2)**

| **Sequence ID** | **Host** | **Location** | **Complete**  **genome Nt** | **AV1** | | **AV2** | | **AC1** | | **AC2** | | **AC3** | | **AC4** | |
| --- | --- | --- | --- | --- | --- | --- | --- | --- | --- | --- | --- | --- | --- | --- | --- |
|  |  |  |  | **Nt** | **aa** | **Nt** | **aa** | **Nt** | **aa** | **Nt** | **aa** | **Nt** | **aa** | **Nt** | **aa** |
| MW917145 | Urdbean | India | 98.7 | 98.3 | 99.6 | 96.7 | 93.8 | 99.7 | 99.4 | 98.2 | 95.3 | 98.2 | 98.5 | 99.3 | 97.9 |
| KP779635 | Frenchbean | India | 98.5 | 98 | 99.6 | 98.5 | 97.3 | 99.1 | 98.2 | 96.9 | 93.3 | 96.5 | 94.7 | 99.6 | 98.9 |
| MH324445 | Soybean | India | 98.3 | 98 | 99.6 | 98.5 | 97.3 | 99.1 | 98.2 | 96.9 | 93.3 | 96.5 | 94.7 | 99.6 | 98.9 |
| KR052025 | Soybean | India | 98 | 97.9 | 80.5 | 98.5 | 96.4 | 98.2 | 95.8 | 96.2 | 93.3 | 97.5 | 97 | 97.3 | 96.9 |
| KX363947 | Pigeonpea | India | 97.6 | 98.3 | 99.6 | 97.3 | 95.5 | 97.7 | 94 | 96.4 | 94.6 | 97.5 | 97 | 97 | 94.9 |
| FN794200 | Frenchbean | India | 97.4 | 99 | 99.6 | 99.1 | 98.2 | 96.5 | 92.9 | 97.7 | 94.6 | 96.5 | 94.7 | 96.3 | 92.9 |
| MZ235792 | Blackgram | India | 97.2 | 98.7 | 98.8 | 98.2 | 97.3 | 96.5 | 92.3 | 97.5 | 94 | 96.2 | 95.5 | 96.6 | 92.9 |
| AJ512495 | Mungbean | Pakistan | 97.2 | 98.4 | 99.2 | 98.8 | 98.2 | 96.6 | 92.8 | 96.9 | 94 | 97.7 | 97 | 95.6 | 90.9 |
| MW600934 | Soybean | India | 96.7 | 96.8 | 94.1 | 98.5 | 98.2 | 96.6 | 93.1 | 97.5 | 94 | 96 | 95.5 | 96.3 | 92.9 |
| DQ389154 | Cowpea | India | 96.1 | 96.5 | 97.6 | 94.4 | 85.8 | 97.2 | 93.7 | 94.9 | 93.3 | 96.2 | 94 | 97.3 | 94.9 |
| MF683072 | Tomato | India | 96.1 | 97 | 98.8 | 95.9 | 89.3 | 97.1 | 94 | 95.8 | 91.3 | 95.5 | 93.2 | 97.3 | 96.9 |
| KU950430 | Mungbean | India | 96.1 | 96.5 | 98.4 | 96.7 | 92 | 96.9 | 93.4 | 96.2 | 92 | 95.8 | 94.7 | 97 | 96.9 |
| OK431083 | Mungbean | India | 96 | 96.5 | 97.6 | 96.1 | 91.1 | 96.8 | 93.1 | 97.5 | 96 | 95.8 | 92.5 | 96.3 | 93.9 |
| HF922628 | Soybean | India | 96 | 95.9 | 97.6 | 95.6 | 89.3 | 96.8 | 93.8 | 94 | 91.3 | 96.5 | 94.7 | 96.3 | 86.8 |
| AY547317 | Dolichos | India | 95.4 | 96.6 | 98.4 | 94.4 | 85.8 | 97.3 | 94 | 94.9 | 92.6 | 93.8 | 90.2 | 97.3 | 94.9 |
| KC019304 | Frenchbean | India | 95.7 | 96.5 | 97.6 | 94.7 | 86.7 | 96.9 | 93.1 | 94.7 | 92 | 93.5 | 89.5 | 96.6 | 93.9 |
| MT027035 | Urdbean | India | 95.5 | 96.6 | 97.6 | 95.6 | 91.1 | 96.8 | 93.7 | 94.7 | 90 | 92.5 | 88.8 | 96 | 93.9 |
| AY271895 | Mungbean | Nepal | 95.4 | 96.3 | 98 | 94.7 | 87.6 | 96.6 | 92.6 | 94.7 | 93.3 | 94.5 | 91.7 | 95.6 | 90.9 |
| AJ512498 | Mungbean | Pakistan | 95 | 96.3 | 97.6 | 94.4 | 85.8 | 96.2 | 91.4 | 93.3 | 90 | 93.5 | 91 | 96 | 91.9 |
| JN543395 | Kidneybean | Nepal | 94.9 | 96.5 | 98.4 | 94.4 | 85.8 | 73.1 | 69.1 | 92 | 90.6 | 94 | 90.2 | 97.3 | 94.9 |
| MK757218 | Cucumber | Oman | 94.7 | 94.5 | 96.8 | 89.9 | 83 | 95.1 | 90.2 | 95.8 | 92.6 | 94.5 | 91 | 95 | 87.8 |
| JN368437 | Yardlongbean | Indonesia | 94.7 | 95.4 | 97.6 | 93.5 | 86.7 | 94.9 | 89.9 | 95.3 | 90.6 | 95 | 92.5 | 94.6 | 88.8 |
| AY618902 | Cowpea | India | 94.4 | 94.7 | 96.5 | 94.4 | 88.4 | 94.7 | 88.4 | 95.8 | 92.6 | 91.8 | 88.8 | 93 | 85.8 |
| KX452227 | Frenchbean | Oman | 94.5 | 94.4 | 97.2 | 90.1 | 83 | 95 | 90.2 | 96 | 92.6 | 94 | 90.2 | 94.6 | 86.8 |
| KX671566 | Soybean | Pakistan | 95.1 | 96.1 | 99.2 | 94.1 | 84.9 | 96 | 90.5 | 94.9 | 92 | 94.5 | 91.7 | 94.6 | 87.8 |
| AY937195 | Mungbean | India | 94.7 | 94.7 | 97.2 | 94.1 | 87.6 | 95.4 | 90.8 | 96 | 93.3 | 89.4 | 80 | 95.3 | 87.8 |
| KX711621 | Soybean | Pakistan | 94.9 | 95.8 | 98.8 | 93.8 | 84 | 95.9 | 90.5 | 94.2 | 91.3 | 94 | 91 | 92.6 | 85.8 |
| OK431079 | Mungbean | India | 96 | 96.8 | 98 | 96.7 | 92 | 96.1 | 91 | 96.2 | 92.6 | 95.8 | 94.7 | 95 | 93.9 |
| AM932427 | Limabean | India | 82.3 | 82.9 | 86.4 | 79.5 | 74 | 83.2 | 81.8 | 82.3 | 78.9 | 82 | 80.1 | 81.2 | 78.7 |
| AY309241 | Dolichos | India | 62.7 | 55.1 | 57.4 | 61.2 | 47.6 | 25.6 | 14.8 | 56.7 | 39.9 | 57.7 | 39.4 | 42.1 | 15.5 |
| HQ264185 | Tomato | India | 62.1 | 53.3 | 18.7 | 42.4 | 62.1 | 11.9 | 28.7 | 52.3 | 56.3 | 56.7 | 41.2 | 39.5 | 13.4 |
