# Supplementary material for: Development of infectious clones of mungbean yellow mosaic India virus (MYMIV, Begomovirus vignaradiataindiaense) infecting mungbean [Vigna radiata (L.) R. Wilczek] and evaluation of a RIL population for MYMIV resistance
Source: PLoS One. 2024 Oct 22;19(10):e0310003. doi: 10.1371/journal.pone.0310003 (PMC11495560; doi:10.1371/journal.pone.0310003)
Supplement: S2 Table — (DOCX) [file pone.0310003.s009.docx]

**S2 Table. Sequence identity of the complete DNA B genome, different ORFs at nucleotide (Nt) and amino acid (aa) level of begomovirus clone with other related begomoviruses (analyzed by Mega X version 10.2.6 and BioEdit version 7.2)**

| **Sequence ID** | **Host** | **Location** | **Complete**  **genome Nt** | **BV1** | | **BC1** | |
| --- | --- | --- | --- | --- | --- | --- | --- |
|  |  |  |  | **Nt** | **Aa** | **Nt** | **Aa** |
| MW917146 | Urdbean | India | 95.8 | 96.3 | 98 | 96.4 | 98.6 |
| KX363948 | Pigeonpea | India | 95.6 | 96.1 | 97.6 | 96 | 98.3 |
| MH577014 | Tomato | India | 95.18 | 96.6 | 96 | 96 | 98.3 |
| KP779634 | Frenchbean | India | 95.18 | 96.4 | 95.7 | 95.5 | 97.9 |
| MF693402 | Pigeonpea | India | 95.1 | 96.1 | 98 | 94.8 | 98.3 |
| FR714861 | Frenchbean | India | 95 | 96.3 | 96.8 | 95.5 | 97.6 |
| MH324446 | Soybean | India | 94.8 | 96.6 | 96 | 95 | 98.9 |
| AY271894 | Mungbean | India | 94.7 | 97.2 | 97.6 | 95.8 | 97.3 |
| EU523046 | Soybean | India | 94.4 | 95.8 | 98 | 95.8 | 97.3 |
| KP828155 | Soybean | India | 93.7 | 95.9 | 98 | 95.9 | 98.3 |
| FM202440 | Mungbean | Pakistan | 93.9 | 95.7 | 97.2 | 94.7 | 99.3 |
| AJ420331 | Soybean | India | 93.7 | 95.7 | 95.7 | 95.6 | 98.3 |
| DQ061273 | Dolichusbean | India | 93.4 | 95.3 | 96.4 | 96 | 98.3 |
| JN543396 | Kidney bean | Nepal | 93.4 | 94.9 | 97.2 | 94.5 | 98.6 |
| MT232630 | Soybean | India | 93.1 | 94.8 | 96.4 | 93.7 | 93.6 |
| MW659819 | Soybean | India | 92.9 | 94.4 | 94.5 | 95.6 | 98.3 |
| MN698290 | Mothbean | India | 92.2 | 95.9 | 97.2 | 93 | 93.9 |
| MZ356197 | Blackgram | India | 91 | 93.9 | 96 | 93.5 | 96.6 |
| OK431080 | Mungbean | India | 90.8 | 93.7 | 96 | 93.4 | 96.3 |
| MF683073 | Tomato | India | 90.7 | 94 | 96.8 | 93 | 96.6 |
| KU950431 | Mungbean | India | 90.6 | 93.9 | 96.4 | 93.3 | 94.9 |
| KP319017 | Mungbean | India | 90.4 | 93.5 | 96.8 | 91.8 | 94.2 |
| MK757224 | Tomato | Oman | 89.5 | 94.1 | 96.4 | 92.5 | 96.3 |
| DQ865202 | Mothbean | India | 89 | 93.5 | 96.4 | 91.3 | 94.9 |
| MZ130504 | Blackgram | India | 88.2 | 93.6 | 96 | 88 | 88.8 |
| AM992617 | Mungbean | Pakistan | 94.8 | 96.8 | 96.8 | 95.9 | 97.9 |
| JQ327848 | limabean | Nepal | 93.4 | 85.3 | 87.3 | 95.6 | 99.3 |
| MN885470 | Soybean | Pakistan | 92.4 | 93.7 | 94.5 | 96 | 98.3 |
| AM932430 | Limabean | India | 68.1 | 48.6 | 50.4 | 65.3 | 52.2 |
| KJ481206 | Dolichos | India | 55.1 | 43.2 | 55.6 | 51 | 46.4 |
| HQ264186 | Tomato | India | 45.1 | 21.5 | 33 | 14.3 | 37.2 |
