# Supplementary material for: Development of infectious clones of mungbean yellow mosaic India virus (MYMIV, Begomovirus vignaradiataindiaense) infecting mungbean [Vigna radiata (L.) R. Wilczek] and evaluation of a RIL population for MYMIV resistance
Source: PLoS One. 2024 Oct 22;19(10):e0310003. doi: 10.1371/journal.pone.0310003 (PMC11495560; doi:10.1371/journal.pone.0310003)
Supplement: S3 Table — (DOCX) [file pone.0310003.s010.docx]

**S3 Table. List of functional motifs identified in the amino acid sequence of different ORFs in MYMIV (identified by Motif Scan tool)**

| **DNA A** | | | | | |
| --- | --- | --- | --- | --- | --- |
| **ORFs** | **Motifs** | **Position in**  **amino acid sequence** | **ORFs** | **Motifs** | **Position in amino**  **acid sequence** |
| AV1 | Amidation site | 106-109 | AC2 | N-glycosylation site | 100-103 |
|  | N-glycosylation site | 131-134, 184-187,  222-225 |  | Casein kinase II phosphorylation site | 45-48, 76-79, 122-125 |
|  | cAMP- and cGMP-dependent protein  kinase phosphorylation site | 43-46 |  | N-myristoylation site | 108-113 |
|  | Casein kinase II phosphorylation site | 149-152 |  | Protein kinase C  phosphorylation site | 29-31, 67-69 |
|  | N-myristoylation site | 34-39, 99-104,  154-159, 189-194 |  | GETHR pentapeptide repeat | 65-79 |
|  | Protein kinase C  phosphorylation site | 46-48, 87-89, 103-105,  118-120, 168-170, 244-246 |  | Geminivirus AL2 protein | 15-148 |
|  | Bipartite nuclear localization  signal profile | 41-55 | AC3 | N-myristoylation site | 61-66, 80-85, |
| AV2 | Geminivirus V1 protein | 1-78 |  | Protein kinase C  phosphorylation site | 28-30 |
| AC1 | N-glycosylation site | 168-171, 357-360 | AC4 | cAMP- and cGMP-dependent  protein kinase phosphorylation site | 19-22 |
|  | ATP/GTP-binding site  motif A (P-loop). | 221-228 |  | Casein kinase II  phosphorylation site | 28-31, 68-71, 88-91 |
|  | Casein kinase II phosphorylation site | 24-27, 96-99, 102-105, 129-132, 142-145, 198-201, 310-313, 335-338 |  | N-myristoylation site | 21-26 |
|  |  |  |  | Protein kinase C  phosphorylation site | 13-15, 17-19, 37-39,  40-42, 53-55, 84-86, 93-95 |
|  | N-myristoylation site | 125-130, 295-300, 347-352 |  | Geminivirus C4 protein | 3-87 |
|  | Protein kinase C  phosphorylation site | 122-124, 225-227, 310-312, 335-337 |  |  |  |
|  | Tyrosine kinase  phosphorylation site | 95-103 |  |  |  |
|  | Geminivirus Rep  catalytic domain | 7-120 |  |  |  |
|  | Geminivirus rep  protein central domain | 126-230 |  |  |  |
| **DNA B** | | | | | |
| **ORFs** | **Motifs** | **Position in amino acid sequence** | **ORFs** | **Motifs** | **Position in amino acid sequence** |
| BV1 | Casein kinase II phosphorylation site | 55-58 | BC1 | N-glycosylation site | 3-6, 235-238 |
|  | Protein kinase C phosphorylation site | 9-11 |  | Casein kinase II phosphorylation site | 24-27, 36-39, 74-77, 109-112, 114-117, 225-228, 231-234, 237-240, 271-274 |
|  | Ubiquitin family | 6-11, 6-74 |  |  |  |
|  |  |  |  | N-myristoylation | 6-11, 130-135 |
|  |  |  |  | Protein kinase C phosphorylation | 74-76, 109-111, 144-146, 221-223, 289-291 |
|  |  |  |  | Aldose 1-epimerase | 195-217 |
|  |  |  |  | Geminivirus BL1 movement protein | 12-298 |
