# Supplementary material for: Development of infectious clones of mungbean yellow mosaic India virus (MYMIV, Begomovirus vignaradiataindiaense) infecting mungbean [Vigna radiata (L.) R. Wilczek] and evaluation of a RIL population for MYMIV resistance
Source: PLoS One. 2024 Oct 22;19(10):e0310003. doi: 10.1371/journal.pone.0310003 (PMC11495560; doi:10.1371/journal.pone.0310003)
Supplement: S4 Table — (DOCX) [file pone.0310003.s011.docx]

**S4 Table. Identification of nuclear localization signal (NLS) in different ORFs of MYMIV clone.**

| **Name of the virus** | **ORFs** | **Position amino acid sequence** | **Amino acid sequence** | **Score** |
| --- | --- | --- | --- | --- |
| **MYMIV DNA A** | AV1 | 36 | VPTNMKRRRWT | 8 |
|  |  | 37 | PTNMKRRRWT | 6 |
|  |  | 14 | ISNARRRLNFDTPLMLPASAGGVPTNMKRRRW | 5.3 |
|  | AC1 | 17 | TYPKCPLTKEDALEQLLALSTPVNKKFIRI | 5.7 |
|  |  | 17 | TYPKCPLTKEDALEQLLALSTPVNKKFIRI | 6.7 |
| **MYMIV DNA B** | BV1 | 198 | PLSRRKCPLWTTFKDPDQGNCGGNYKNIS | 5.4 |
|  | BC1 | 150 | DIRFKPPTINILSKDYTADCVDFWSVEKPKPI | 3 |
|  |  | 246 | RHLHKLPEASLDPGDSISQTQSNAMSKREIED | 3.1 |
