# Supplementary material for: Development of infectious clones of mungbean yellow mosaic India virus (MYMIV, Begomovirus vignaradiataindiaense) infecting mungbean [Vigna radiata (L.) R. Wilczek] and evaluation of a RIL population for MYMIV resistance
Source: PLoS One. 2024 Oct 22;19(10):e0310003. doi: 10.1371/journal.pone.0310003 (PMC11495560; doi:10.1371/journal.pone.0310003)
Supplement: S5 Table — (DOCX) [file pone.0310003.s012.docx]

**S5 Table. Recombination analysis of DNA A of Mungbean yellow mosaic India virus using RDP 4.101 tool showing intraspecific recombination**

| **Breakpoints** | **Position in genome** | **Avg, P-value of the**  **recombination event** | **Major parent** | **Minor parent** |
| --- | --- | --- | --- | --- |
| 1450-1859 nt | AC2, AC3 | RDP (> 1.0); BootScan (1.238 x10^-02^);  MaxChi (1.156 x 10^-01^); Chimaera (2.547 x 10^-01^); SiScan (1.031x 10^-2^);  3Seq (9.365 x 10^-02^) | KX363947_MYMIV- IARI,  New Delhi-Pigeonpea | FN794200_MYMIV-IHBT, Palampur-Frenchbean |
