# Supplementary material for: Development of infectious clones of mungbean yellow mosaic India virus (MYMIV, Begomovirus vignaradiataindiaense) infecting mungbean [Vigna radiata (L.) R. Wilczek] and evaluation of a RIL population for MYMIV resistance
Source: PLoS One. 2024 Oct 22;19(10):e0310003. doi: 10.1371/journal.pone.0310003 (PMC11495560; doi:10.1371/journal.pone.0310003)
Supplement: S6 Table — (DOCX) [file pone.0310003.s013.docx]

**S6 Table. Recombination analysis of DNA B of Mungbean yellow mosaic India virus using RDP 4.101 tool showing intraspecific recombination**

| **Breakpoints** | **Position in genome** | **Avg, P-value of the recombination event** | **Major parent** | **Minor parent** |
| --- | --- | --- | --- | --- |
| 185-346 nt | Intergenic region | RDP (3.144 x 10^-01^); GENECONV (1.008 x 10^-06^); MaxChi (1.373 x 10^-03^); Chimaera (1.251 x 10^-03^); SiScan (5.428 x 10^-04^); 3Seq (1.571 x 10^-03^) | AM992617_MYMIV- Pakistan- Mungbean | MW659819_MYMIV Lalitpur-Uttar Pradesh- Soybean |
