# Supplementary material for: Development of infectious clones of mungbean yellow mosaic India virus (MYMIV, Begomovirus vignaradiataindiaense) infecting mungbean [Vigna radiata (L.) R. Wilczek] and evaluation of a RIL population for MYMIV resistance
Source: PLoS One. 2024 Oct 22;19(10):e0310003. doi: 10.1371/journal.pone.0310003 (PMC11495560; doi:10.1371/journal.pone.0310003)
Supplement: S7 Table — (DOCX) [file pone.0310003.s014.docx]

**S7 Table. Preliminary screening and grouping of 175 RILs based on their reaction to MYMIV in *Kharif* 2020**

| **Disease reaction** | **No. of RILs** | **PDI range (Mean)** | **PDS range (Mean)** | **CI range (Mean)** | **AUDPC range** |
| --- | --- | --- | --- | --- | --- |
| Highly resistant (HR) | 3 | 7-15.6(12.2) | 8.4-17.9(14.74) | 3.4-4.04(3.7) | 78.9-117.4 |
| Resistant (R) | 18 | 1.7-35.8(16.7) | 17.8-44.3(27.9) | 4.3-8.9(7.1) | 115.3-1035 |
| Moderately resistant (MR) | 37 | 16.1-50.9(29.3) | 20.5-78.9(44.8) | 9.3-21.7(14.4) | 164.3-748.3 |
| Moderately susceptible (MS) | 75 | 23.5-72.5(43) | 25.2-83.1(51.6) | 19.6-38.5(26.5) | 144.1-1415.3 |
| Susceptible (S) | 35 | 39.7-86.2(61) | 51.9-95.2(71.3) | 39.3-68.3(50.7) | 427.1-1840 |
| Highly susceptible (HS) | 7 | 70.6-84.2(75.7) | 75.4-91.1(81.8) | 70.6-84.2(74.9) | 820.3-1825.6 |
