# Supplementary material for: Development of infectious clones of mungbean yellow mosaic India virus (MYMIV, Begomovirus vignaradiataindiaense) infecting mungbean [Vigna radiata (L.) R. Wilczek] and evaluation of a RIL population for MYMIV resistance
Source: PLoS One. 2024 Oct 22;19(10):e0310003. doi: 10.1371/journal.pone.0310003 (PMC11495560; doi:10.1371/journal.pone.0310003)
Supplement: S8 Table — (DOCX) [file pone.0310003.s015.docx]

**S8 Table. Advance field screening and grouping of 175 RILs based on their reaction to MYMIV in *Kharif* 2021**

| **Disease reaction** | **No. of RILs** | **PDI range (Mean)** | **PDS range (Mean)** | **CI range (Mean)** | **AUDPC range** |
| --- | --- | --- | --- | --- | --- |
| Highly resistant (HR) | 3 | 7-13.7(9.7) | 16.9-21.9(18.6) | 3.8-4(3.9) | 56.9-129.3 |
| Resistant (R) | 19 | 10.6-53.9(20.5) | 15.5-49(29) | 5.3-8.7(6.9) | 83.6-289.9 |
| Moderately resistant (MR) | 26 | 16-52.9(31) | 20.3-87.8(42.9) | 9.4-19.9(15.5) | 173.2-787.7 |
| Moderately susceptible (MS) | 71 | 24.1-85.7(46) | 17.1-89.6(51.9) | 19.5-38.7(28.2) | 317.8-1139.3 |
| Susceptible (S) | 39 | 41.5-91.9(64.5) | 28.2-100(65.6) | 40.4-68.3(54.8) | 603.5-1698.7 |
| Highly susceptible (HS) | 17 | 41.8-100(75.3) | 51.8-100(75.7) | 70.4-86.6(73.7) | 650-1656.3 |
