# Supplementary material for: Development of infectious clones of mungbean yellow mosaic India virus (MYMIV, Begomovirus vignaradiataindiaense) infecting mungbean [Vigna radiata (L.) R. Wilczek] and evaluation of a RIL population for MYMIV resistance
Source: PLoS One. 2024 Oct 22;19(10):e0310003. doi: 10.1371/journal.pone.0310003 (PMC11495560; doi:10.1371/journal.pone.0310003)
Supplement: S9 Table — (DOCX) [file pone.0310003.s016.docx]

**Table S9. The cycle threshold (C_t_) values of replicate assays for MYMIV.**

| **Serial No** | **Sample Name** | **Target Name** | **C_t_ values^a^** | **SD^b^** | **SE^c^** | **CV^d^** |
| --- | --- | --- | --- | --- | --- | --- |
| 1 | PBAB3.1 | AV1 | 16.07 | 0.56 | 0.32 | 3.50 |
|  | ACT | AV1 | 20.29 | 0.02 | 0.01 | 0.12 |
| 2 | PBA2.1 | AV1 | 28.01 | 0.71 | 0.41 | 2.55 |
|  | ACT | AV1 | 20.40 | 0.47 | 0.27 | 2.32 |
| 3 | PMRB1.1 | AV1 | 24.17 | 0.13 | 0.07 | 0.52 |
|  | ACT | AV1 | 28.84 | 1.07 | 0.61 | 3.69 |
| 4 | PMRA2 | AV1 | 25.80 | 0.46 | 0.27 | 1.78 |
|  | ACT | AV1 | 20.58 | 0.43 | 0.25 | 2.09 |
| 5 | RIL10A2 | AV1 | 29.19 | 0.32 | 0.18 | 1.08 |
|  | ACT | AV1 | 21.24 | 0.19 | 0.11 | 0.91 |
| 6 | RIL10B3 | AV1 | 19.83 | 0.19 | 0.11 | 0.96 |
|  | ACT | AV1 | 20.43 | 0.30 | 0.17 | 1.47 |
| 7 | RIL13B4.1 | AV1 | 25.26 | 0.17 | 0.10 | 0.67 |
|  | ACT | AV1 | 28.95 | 0.99 | 0.57 | 3.43 |
| 8 | RIL13A5 | AV1 | 29.18 | 0.11 | 0.06 | 0.36 |
|  | ACT | AV1 | 23.16 | 1.81 | 1.04 | 7.81 |
| 9 | RIL21B.2 | AV1 | 23.99 | 0.94 | 0.54 | 3.92 |
|  | ACT | AV1 | 21.57 | 0.31 | 0.18 | 1.43 |
| 10 | RIL21A2.1 | AV1 | 23.99 | 0.94 | 0.54 | 3.92 |
|  | ACT | AV1 | 21.57 | 0.31 | 0.18 | 1.43 |
| 11 | RIL92B2S | AV1 | 14.93 | 0.84 | 0.49 | 5.64 |
|  | ACT | AV1 | 22.22 | 0.90 | 0.52 | 4.07 |
| 12 | RIL92A1 | AV1 | 27.97 | 0.56 | 0.32 | 2.01 |
|  | ACT | AV1 | 22.14 | 0.32 | 0.18 | 1.44 |
| 13 | RIL123B1.1 | AV1 | 22.77 | 0.75 | 0.44 | 3.32 |
|  | ACT | AV1 | 25.30 | 0.17 | 0.10 | 0.67 |
| 14 | RIL123A5 | AV1 | 29.51 | 0.60 | 0.34 | 2.02 |
|  | ACT | AV1 | 23.16 | 1.81 | 1.04 | 7.81 |
| 15 | RIL131B1.1 | AV1 | 22.61 | 0.54 | 0.31 | 2.37 |
|  | ACT | AV1 | 28.81 | 0.50 | 0.29 | 1.72 |
| 16 | RIL131A5 | AV1 | 28.23 | 0.52 | 0.30 | 1.85 |
|  | ACT | AV1 | 22.47 | 0.56 | 0.32 | 2.47 |
| 17 | RIL163B4.1 | AV1 | 24.47 | 0.66 | 0.38 | 2.68 |
|  | ACT | AV1 | 29.10 | 0.92 | 0.53 | 3.16 |
| 18 | RIL163A2 | AV1 | 30.43 | 1.18 | 0.68 | 3.88 |
|  | ACT | AV1 | 21.28 | 0.21 | 0.12 | 1.00 |
| 19 | PBS(Calibrator) | AV1 | 9.61 | 0.82 | 0.47 | 8.52 |
|  | ACT | AV1 | 20.21 | 0.42 | 0.24 | 2.05 |

^a^The C_t_ value represents the mean of the three replicates of that experiment's SYBR Green PCR run. ^b^Standard deviation; ^c^Standard Error; ^d^Coefficient of Variation
